# Cladistic analysis and redefinition of the *Dasybasis* Macquart *s. str.* (Diptera: Tabanidae) in the Neotropical region

**DOI:** 10.1590/0074-02760240245

**Published:** 2025-02-24

**Authors:** Christian R González, Daniel Rafael Miranda-Esquivel

**Affiliations:** 1 Universidad Metropolitana de Ciencias de la Educación, Facultad de Ciencias Básicas, Instituto de Entomología, Santiago, Chile; 2 Universidad Industrial de Santander, Escuela de Biología, Laboratorio de Sistemática y Biogeografía, Bucaramanga, Santander, Colombia

**Keywords:** Diachlorini, horse flies, phylogeny, taxonomy

## Abstract

**BACKGROUND:**

The works of Lutz & Neiva, published 115 years ago in the Memórias do Instituto Oswaldo Cruz, are pioneering for the study of Neotropical Tabanidae. These studies emphasised the importance of biological collections and the description of species from the exploration of South American areas. *Dasybasis* Macquart, 1847 has traditionally been considered a large genus of tabanids restricted to the Australasian, Neotropical, and Andean regions. *Dasybasis* species exhibit a high degree of morphological similarity, making specific differentiation challenging. Moreover, some of these features are also present in other taxa, suggesting that they may not be homologous characters and should not be used to define the genus.

**OBJECTIVES:**

This study aimed to assess the monophyly of *Dasybasis* and establish its major monophyletic groups.

**METHODS:**

We conducted an implied weighting analysis using morphological characters, and wing landmarks from 91 terminal species.

**FINDINGS:**

For the total evidence analyses, aligning with either *Tabanus* Linnaeus or *Dasybasis appendiculata* Macquart yielded slightly different trees. Classical morphology and total evidence topology aligned with *D. appendiculata* are the same, while differing from the total evidence topology aligned with *Tabanus* in two nodes.

**MAIN CONCLUSIONS:**

Our results indicate that *Dasybasis* was not a monophyletic group, and that this name should be restricted to species with a distribution in Australasia; while Neotropical *Dasybasis* species are recovered in different clades. The genera *Archiplatius*, *Pseudoselasoma*, and *Stypommia* are revalidated. This study provides a revised phylogenetic framework for “*Dasybasis*” and related taxa, highlighting the need for a more nuanced understanding of morphological character evolution within the tribe Diachlorini.

Since the beginning of entomology in ancient Greece,^1^ a major concern has been to describe the most characteristic insects found in different countries and to maintain representative collections of these insects, with the monophyly of the taxa as a paramount idea.^2^

In the literature on the study of Neotropical Tabanidae, the pioneering works of Lutz & Neiva^3^ ^,^ ^4^ are among the first efforts. They emphasised the importance of biological collections and the description of species from the exploration of new regions of our continent, particularly the vast territory of Brazil. These two Brazilian scientists made significant contributions to the field of medical entomology, developing much of their entomological work at the Oswaldo Cruz Institute and being active contributors to the journal “Memórias do Instituto Oswaldo Cruz” since its inception in 1909. Their contributions included two papers, “*Erephosis auricincta*. Uma nova motuca da subfamilia: Panogoniinae”^3^ and “Contribuicões para o conhecimento da fauna indígena de Tabanidas”.^4^ The work of Lutz & Neiva^3^ helped break the trend of the nineteenth-century European scientists describing neotropical species from biological material collected by expeditions that travelled through our continent in the early nineteenth century.^5^

The first description of a Brazilian horse flies species was made by Fabricius,^6^ but Wiedemann^7^ ^,^ ^8^ ^,^ ^9^ ^,^ ^10^ made the greatest contributions to the knowledge of the family. Lutz^11^ ^,^ ^12^ ^,^ ^13^ ^,^ ^14^ and Lutz & Neiva^3^ ^,^ ^4^ initiated the description of tabanid species by Brazilian scientists and emphasised the importance of biological collections in understanding the natural history of our countries. This is reflected in the nascent collection of Tabanidae, which began to be formed at the Oswaldo Cruz Institute, as highlighted in their research.^4^ Today, 115 years after Lutz & Neiva,^4^ the tabanid collection of the Oswaldo Cruz Institute (http://ceioc.fiocruz.br/catalogue) has more than 12.000 specimens and 430 primary types (M. Felix, personal comm.), mainly composed of specimens from the collection of Dr Adolph Lutz, bringing together species from Brazil and other South American countries.

The tribe Diachlorini of Tabanidae, which includes more than 60 genera and more than 900 species,^15^ is cosmopolitan, although it exhibits greater diversity in Australasian (14 genera)^16^ and Neotropical (39 genera) regions.^17^ ^,^ ^18^ The biology and knowledge of the immature stages of the vast majority of the more than 560 Neotropical species of Diachlorini are unknown, making a total morphology analysis not feasible at the moment. The data gathered so far indicates some unusual feeding habits, such as *Leucotabanus* Lutz larvae living in association with termites, while others live in association with bromeliads (*Stibasoma* Schiner species).

The genus *Dasybasis* was erected by Macquart, 1847 as a monotypic genus, including the Australian species *Dasybasis appendiculata* (Fig. 1A-E). The genus represents one of the most diverse groups within the southern Neotropical fauna, comprising more than 70 valid taxa.^17^ The Neotropical species of the genus have been extensively investigated by different researchers, such as Brèthes,^19^ Enderlein,^20^ Kröber,^21^ Coscarón & Philip,^22^ González.^23^ *Dasybasis* is also present in Australia and New Zealand, with 73 species described in two subgenera,^16^ as well as in New Caledonia^24^ and the Fiji Archipelago.^25^ The genus exhibits a widespread distribution in southern Argentina and Chile, with 47 and 34 species, respectively. It is classified as the evolutionarily oldest group within the tribe Diachlorini, predominantly occurring in the colder zones of the Neotropics in specialised habitats.^26^ ^,^ ^27^

The description of the species has been frequently based on females, which, due to their hematophagous habit, are more frequently collected, and for the vast majority of species, both males and immature stages are unknown. Coscarón & Philip^22^ defined *Dasybasis* without any formal analysis, using mainly external adult morphological characters, predominantly female. However, most of these characters are variable and sometimes difficult to evaluate, such as the basicosta, ocular ommatrichia, the shape of the basal callus, or the appendix on R_4_.

Different *Dasybasis* species exhibit variation in certain morphological features, and the same states are also present in other taxa, which may indicate that they might not be homologous characters and should not be used to support genus classification without formal analysis. For example, ommatrichia is also found in *Eristalotabanus* Kröber, *Protodasyapha* Enderlein; ocellar triangle vestigial in *Querbetia* Fairchild; basal callus as wide as the frons in *Acanthocera* (*Polistimima*) Fairchild, *Dichelacera* (*Dichelacera*) Macquart, and *Stenotabanus* (*Stenotabanus*) Lutz, and globose in *Bolbodimyia* Bigot and *Querbetia* Fairchild; subcallus not inflated in *Buestanmyia* González; scape not inflated in *Buestanmyia*, *Agelanius*, *Chlorotabanus*, *Myiotabanus* and *Pachyschelomyia* Barreto; pedicel with dorsal prolongation occurs in *Cryptotylus* Lutz, *Stibasoma* Schiner, *Phaeotabanus* Lutz; first flagellomere slightly angulate in *Pachyschelomyia* Barreto, *Erioneura* Barreto, *Stenotabanus* Lutz, *Leucotabanus* Lutz; first flagellomere without dorsal tooth occurs in *Buestanmyia*, *Bolbodimyia, Chlorotabanus*, and *Myiotabanus*; maxillary palpi short and stout as in *Buestanmyia*, *Oopelma* Enderlein, *Stibasoma* Schiner, *Stenotabanus* Lutz; scutum with longitudinal stripes as *Haematopotina* Coscarón & Philip, *Eutabanus* Kröber, and *Diachlorus* Osten Sacken. Wing hyaline and smoke-coloured as in *Stenotabanus* Lutz, *Agelanius* Rondani, *Dicladocera* Lutz, appendix on R_4_ as in *Phaetabanus* Lutz, *Dichelacera* (*Orthostyloceras*) Lutz, *Apatolestes* Williston, abdomen with median longitudinal band as in *Stenotabanu*s Lutz, *Haematopotina* Coscarón & Philip, and *Nubiloides* Coscarón & Philip.^28^

The phylogenetic relationships within the genus *Dasybasis* as well as their relationships with other genera remain undetermined or have only been postulated.^22^ Recent taxonomic revisions, not necessarily grounded in phylogenetic analysis, have proposed the elevation of several subgenera of *Dasybasis* to a generic status, including *Agelanius*, *Haematopotina*, *Nubiloides*, and *Scaptiodes*.^23^ Furthermore, several species have been reclassified and transferred between genera. Given the need for a more comprehensive understanding of the group, this study aimed to evaluate the monophyly of the *Dasybasis*, redefine the taxon, establish major monophyletic groups within the clade, and determine the phylogenetic relationships among these groups.

## MATERIALS AND METHODS

*Examined material* - Material from the following individuals were examined: Museo de La Plata (MLP, La Plata, Argentina), Instituto de Entomología de Salta (IES, Salta, Argentina), Canadian National Collection (CNC, Ottawa, Canada), Museo Nacional de Historia Natural (MNHN, Santiago, Chile), Museo de Zoología (MZC, Concepción, Chile), Instituto de Agronomía, (IA, Arica, Chile), Instituto de Entomología, Universidad Metropolitana de Ciencias de la Educación (IEUMCE, Santiago, Chile), Field Museum of Natural History (FMNH, Chicago, USA), Department of Zoology, University of New Hampshire (DZUNH, Durham, USA, Collection Dr Jaime Buestán (Ecuador). The external morphology was examined in dry-pinned specimens, females, and males (when available) for character circumscription. Terminalia were incubated in a 10% KOH solution at 50ºC for 1 h to dissolve the soft tissue, neutralised with acetic acid, rinsed in distilled water, and then dissected in 80% ethanol. Photographs of the flies were taken using a Nikon trinocular stereomicroscope SMZ 1500 and digital camera DS-Fi2. For the general morphology, the depth of the field was enhanced using the NikonTD ACT-2U software by stacking multiple images, whereas the photographs used for landmark data were not enhanced.

*Terminals* - We used 91 terminals, including most of the species of *Dasybasis* (Australasian and Neotropical). As outgroups, we selected at least one, or more species, from *Tabanus*, *Stenotabanus* Lutz, *Agelanius* Rondani, *Haematopotina* Coscarón & Philip, and *Acellomyia* González. The trees were rooted using *Tabanus*. The list of analysed species, additional material examined, and geographical distribution are shown in Supplementary data (Table I).

Morphological characters

**Fig. 1:**
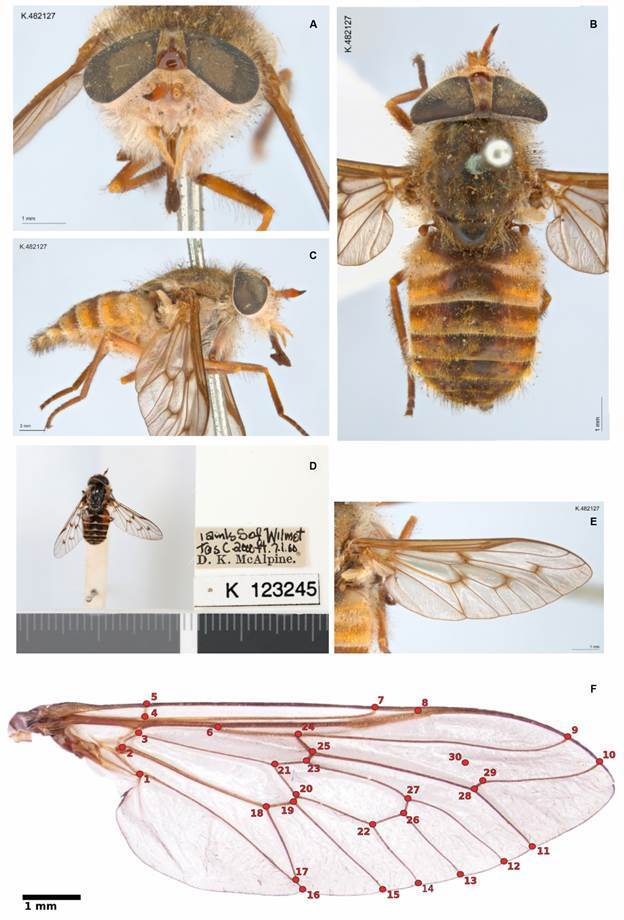
(A-E) habitus of *Dasybasis appendiculata* Macquart, female (K482127 and K123245, ^©^Australian Museum). (A) head in frontal view; (B) head, thorax and abdomen in dorsal view; (C) lateral view; (D) labels; (E) wing; (F) primary landmark points and a semi-landmark, following Torres and Miranda-Esquivel.^30^

*Traditional morphology* - We analysed 58 traditional external morphological characters, including female and male genitalia (Fig. 1A-E). The character states and morphological data matrices are available in Supplementary data (Tables II and III, respectively). The majority of the characters were binary, and we considered the multistate characters to be nonadditive. Species were identified utilising the keys provided by Coscarón & Philip^22^ and by comparison with the type material. The morphological terminology follows Cumming & Wood.^29^

Wing landmarks

We used 29 primary landmark points and one semi-landmark, considering the vein junctions and insertion points. These were selected following Torres & Miranda-Esquivel^30^ (Fig. 1F). We analysed 61 species [Supplementary data (Table IV)]. The dataset was aligned against *Tabanus* or *D. appendiculata* to minimise the sum of the linear distances (see the TNT version 1.6 manual for further details).

Cladistic analysis

Cladistic analysis was conducted using implied weights.^31^ In implied weights, the fit of a character or a tree is defined by the equation: fit = k / (homoplasy + k), where K determines how characters are downweighted according to their homoplasy.^31^ To determine the optimal concavity value, we performed a sensitivity analysis of the classical morphology matrix. We conducted the Jackknife analysis with a fixed cut value of 33% for character deletion against a reference tree (for further details see Goloboff et al.^32^). We tested equal weights and K values from 1 to 70 by using a fast search to guarantee the best fit. The optimal value was defined as the K value that recovered most of the groups scaled to the number of possible nodes. Given the optimal value, a most comprehensive search was conducted for both datasets, the classical morphology and the total evidence, using the xmult / ratchet commands.^33^ ^,^ ^34^ All analyses were carried out using TNT 1.6 (see https://github.com/Dmirandae/DasybasisPhylogeny).^35^

## RESULTS

**Fig. 2:**
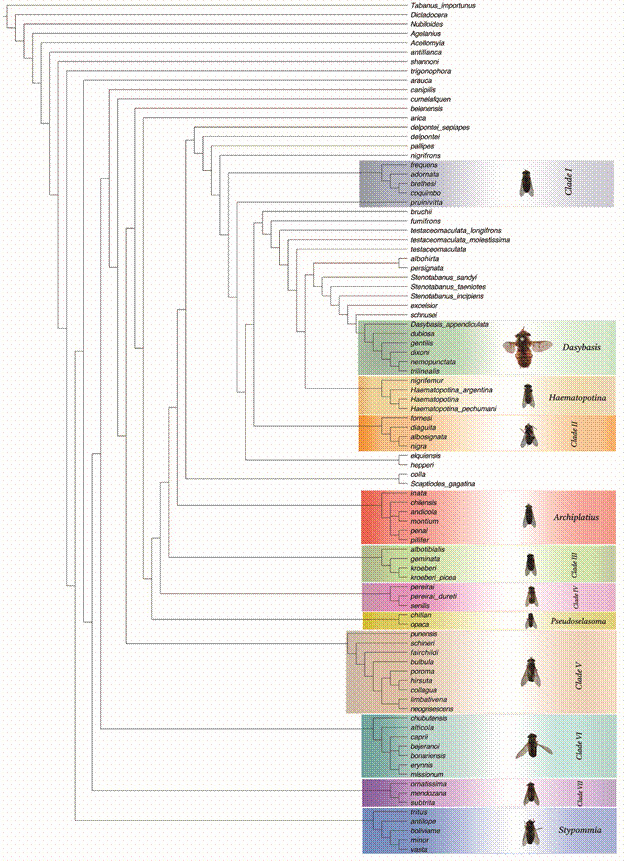
topology for the classical morphology and total evidence aligned with *Dasybasis appendiculata* Macquart under implied weights with a k value of 26.

**Fig. 3:**
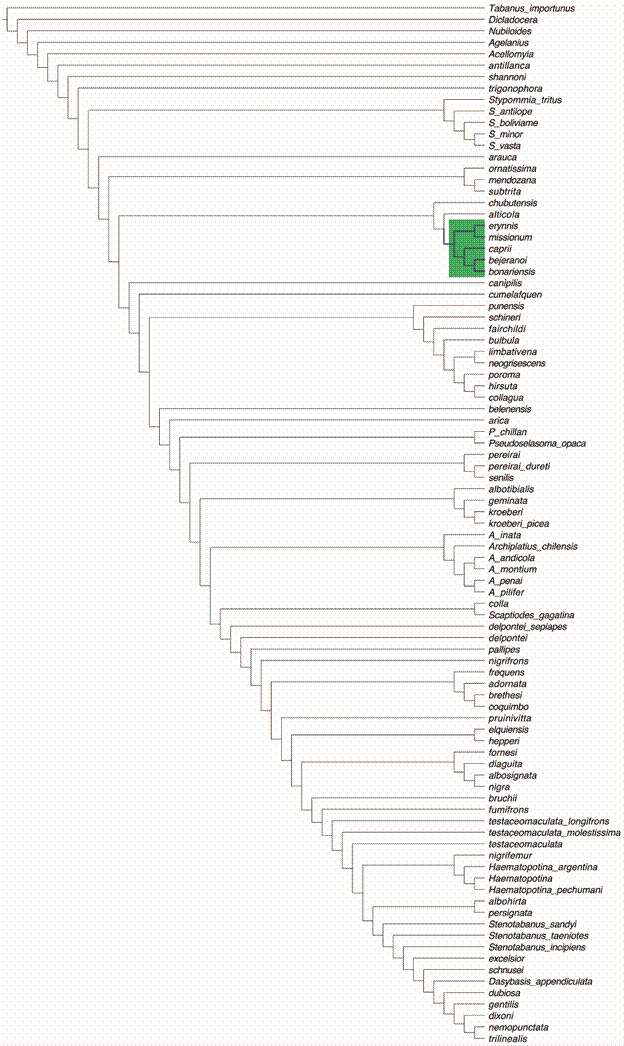
topology for total evidence aligned with *Tabanus* under implied weights with a k value of 26.

We determined the optimal K parameter value to be 26 [Supplementary data (Table V)]. Our analysis yielded two possible topologies: the first one for the classical morphology dataset and total evidence dataset aligned with *D. appendiculata* (Fig. 2. Classical morphology dataset fit = 12.60462. Total evidence fit = 13.415242), and the second one for the total evidence analysis, aligned with *Tabanus* (Fig. 3. Total evidence fit = 13.00150). These two trees differed only in the placement of *caprii* relative to the clades *bejeranoi* + *bonariensis* / *erynnis* + *missionum*. This discrepancy might be attributed to missing landmark data for *bejeranoi*.

Main clades

None of the analyses showed the traditional definition of the *Dasybasis* as a monophyletic group. The name *Dasybasis* should be restricted to the clade containing the type-species of *Dasybasis*, *D. appendiculata*, and the species *D. dubiosa*, *D. gentilis*, *D. dixoni*, *D. nemopunctata*, and *D. trilinealis*. Given the taxa sampled, *Stenotabanus* might not be a monophyletic taxon; therefore, it should be revised, and its current status evaluated. The relationship of *Dasybasis sensu stricto* with *excelsior*, *schnusei*, *Stenotabanus sandyi*, *Stenotabanus incipiens*, and *Stenotabanus taeniotes*, the type species of the genus, could result in the entire group being placed under the same clade, which should be named *Dasybasis*. *Haematopotina* could be considered a monophyletic genus, but the position of *nigrifemur* indicates that the species could be included in *Haematopotina*.

As we could define some clades, we propose the revalidation of the genera:

(1) *Archiplatius* Enderlein *stat. rev.* (type-species *A. chilensis* from Chile) encompasses the following species: *A. inata*, *A. chilensis*, *A. andicola*, *A. montium*, *A. penai*, and *A. pilifer*.

(2) *Pseudoselasoma* Brèthes *stat. rev.* (type-species *P. opaca* from Chile) comprises two species: *P. chillan* and *P. opaca*.

(3) *Stypommia* Enderlein *stat. rev.* (type-species *S. tritus* from Chile) includes the following species: *S. tritus*, *S. antilope*, *S. boliviame*, *S. minor*, and *S. vasta*.

Other clades

In the trees obtained, we can recognise other clades, not formally assigned to a genus name, which we have consecutively named Clades I to VII.

Clade I: *frequens*, *adornata*, *coquimbo* and *brethesi*. The species included in this clade present: ocular ommatrichia, short or long and abundant; pilosity of frons black; basal callus triangular with dorsal-median prolongation, touching the eyes but not subcallus; antenna unicoloured; scape not globose; maxillary palp shorter than head height; appendix on R_4_ absent; base of VIII sternite convex.

Clade II: *fornesi*, *diaguita*, *nigra* and *albosignata*. The species present: frons in vertex concave; basal callus touching eyes and subcallus; ocelli vestigial; antenna unicoloured; appendix on R_4_ present; abdominal terga without median band; female cerci quadrangular; base of VIII sternite convex.

Clade III: *albotibialis*, *geminata*, *kroeberi*, *kroeberi* and *picea*. The species present: ocular band; frons divergent; frontal index up to 2.9; basal callus quadrangular and with dorsal-median prolongation; antenna bicoloured; scape with silver-gray pilosity; appendix on R_4_ present; mid-dorsal abdominal triangles present.

Clade IV: *pereirai*, *pereirai dureti* and *senilis*. The species present: ocular ommatrichia long and abundant; frons divergent; basal callus quadrangular without dorsal-median prolongation; antenna bicoloured; wing hyaline, R_4_ without appendix; base of VIII sternite sinuous.

Clade V: *punensis*, *schineri*, *fairchildi*, *bulbula*, *neogrisescens*, *limbativena*, *poroma*, *collagua* and *hirsuta*. The species have: ocular ommatrichia long and abundant; frons in vertex concave; ocelli vestigial; basal callus quadrangular touching subcallus and eyes and with dorsal-median prolongation; scape globose or semiglobose with whitish pilosity; maxillary palp one third of the proboscis length; wing hyaline or with clouds; vein R_4_ with appendix; abdominal terga without median band.

Clade VI: *chubutensis*, *alticola*, *caprii*, *bejeranoi*, *bonariensis*, *erynnis* and *missionum*. The species have: ocular ommatrichia; antenna bicoloured; scape not globose with short and whitish pilosity; maxillary palp one third of the height of the proboscis; wings hyaline or with clouds; appendix on R_4_ present.

Clade VII: *ornatissima*, *mendozana* and *subtrita*. The species present: ocular ommatrichia scarce, microscopic; frons parallel-sided; frons in vertex concave; basal callus without dorsal-median prolongation; antenna unicoloured; scape not globose, with short pilosity; maxillary palp longer than half of the height of the proboscis; wing with clouds; with mid-dorsal abdominal triangles.

Species

At the species level, while *pereirai* and *kroeberi* could be considered valid species, names such as “*delpontei”* or “*testaceomaculata”* are not a single evolutionary front, as there is no monophyletic taxon grouping the proposed units.

Bootsaping values

Although there are valid transitions at the nodes, the distribution of characters does not warrant higher numerical support. As expected, bootstrapping support for the datasets is low, below 50%.

## DISCUSSION

De Santis^36^ suggested that homoplasy is a common occurrence in evolution and can be more than just “noise” in phylogenetic analysis. In some cases, homoplasy can reveal underlying evolutionary processes, such as convergent evolution, parallel evolution, or reversals, and these patterns often indicate shared developmental or genetic mechanisms, suggesting a deeper common ancestry.

Traditionally, morphological characters have been seen as more homoplastic than other characters, like molecular data. To address this issue within our dataset, we assigned weights to characters based on their homoplasy levels,^31^ thereby improving the accuracy of our phylogenetic reconstructions. This approach recognises that clades can be defined by multiple shared characteristics, therefore providing a more robust framework for phylogenetic analysis. In this scenario, the clades resulted in explicitly defined monophyletic groups according to the homoplasy of the characters. These groups might be polythetic, as they are not necessarily defined by a single character with a unique transformation. Therefore, we defined clades based on a set of transformations that can be unambiguously assigned to a node, allowing for more flexible and biologically meaningful groupings.

When applied to our dataset of wing landmarks and classical morphology, our approach proved effective in addressing homoplasy and providing accurate phylogenetic reconstructions. Although the distribution of characters may not warrant higher numerical bootstrap support, our method offers a robust framework for defining clades and testing the hypotheses.

Wing landmarks have proven useful for differentiating species in Diachlorini^30^ and are congruent with molecular data.^37^ The congruence between classical morphology and landmark data, despite the additive nature of landmark data, provides additional support for our chosen K value. This suggests that our analysis not only provides a compatible solution, but also minimises the assumptions.

The Diachlorini tribe, particularly in the Neotropical region, presents taxonomic challenges due to modified adult morphological characteristics.^28^ Trojan^38^ redefined “Lepidoselagini” (incorrect spelling of Lepiselagini) to include genera previously assigned to Diachlorini and Tabanini, based on characters like head sutures and the presence or absence of an appendix to vein R_4_. However, these characters are highly variable and of limited practical use. Morita et al.^15^ found that posterior probability values within Diachlorini and the distinction made by Trojan^38^ between Lepiselagini and Diachlorini are not informative and result in non-monophyletic groups.

*Dasybasis* was one of the most diverse taxa in the Neotropical region, with more than 70 known species.^17^ ^,^ ^18^ However, *Dasybasis* species show great morphological homogeneity, which makes specific differentiation difficult, with a superficial resemblance to *Hybomitra* and *Tabanus* species.^26^ ^,^ ^27^ The systematics of *Dasybasis* was traditionally based primarily on characters derived from external morphology of adults which creates problems when trying to find enough discrete characters to perform a cladistic analysis, and the definitions of the genus and allies has rendered the so-called groups as non-monophyletic.

The genus in the Neotropical region was revised by Coscarón & Philip,^22^ who divided the genus into five subgenera and characterised it using, among others, the following combination of characters: body colour, eye pilosity, frons width, basal callus shape, ocelli development, maxillary palpi morphology, scape and pedicel shape, flagellomere structure, mesonotum stripes, wing colouration, abdominal markings, and cerci shape. Subsequently, González^23^ elevated *Agelanius* Rondani, *Haematopotina* Coscarón & Philip, *Nubiloides* Coscarón & Philip, and *Scaptiodes* Rondani to the generic level, based on morphological differences from *Dasybasis s. str*.

The morphological homogeneity of tabanids in general and of *Dasybasis* species in particular can be attributed to their adaptation to similar ecological niches along the Andes mountains,^39^ ^,^ ^40^ the azonal vegetation (alpine peatlands), which is found in great extension in the Andes.^41^ The peatlands play a critical role in sustaining a unique diversity of rare and endemic biota in the Andes^42^ to feed on prey and use it as a refuge from the different extreme abiotic factors found along this mountain range. The limited availability of these habitats has restricted the development of diverse physical traits in these insects, leading to a slower rate of morphological evolution. However, this homogeneity at a certain level has been misunderstood, and the use of some characters to define the species of the genus, without cladistic analysis of the grouping, has recycled the same characters to define many genera.

Like other Neotropical Diachlorini genera, *Dasybasis* is a large taxon that groups species that share morphological similarities, although it has not been determined whether they share a common ancestor. Evidently, the affinities and phylogenetic relationships of Diachlorini taxa should be evaluated using tools that can provide better definitions by clearly establishing the boundaries of each unit.^15^

The non-monophyletic nature of *Dasybasis* was not surprising given that the genus itself and its subgenera were weakly diagnosed based on a combination of a few morphological characters. The resulting topology conflicts with the current traditional classification within the *Dasybasis* and Diachlorini. Trojan^38^ resurrected *Pseudoselasoma* and *Archiplatius* to accommodate Neotropical species placed on *Dasybasis*. This facet of his classification was not subsequently recognised.^43^ ^,^ ^23^ Our results agree with those reported by Trojan;^38^ hence, the resurrection of previously synonymised genera will correct the non-monophyletic status within *Dasybasis*, proposing the resurrection of genera from Diachlorini: *Archiplatius*, *Pseudoselasoma*, and *Stypommia*.

Despite the limited numerical support for most branches, our findings provide a phylogenetic framework for *Dasybasis* and its related taxa. Although further analysis may refine the clade composition, it is anticipated that the overall structure will remain stable. Nevertheless, additional studies and sampling should be conducted throughout the range of Neotropical species to identify and describe missing immature stages and verify their taxonomic treatment. This work aims to stimulate research to enhance our understanding of Neotropical tabanids.
