# Supplementary material for: Cladistic analysis and redefinition of the Dasybasis Macquart s. str. (Diptera: Tabanidae) in the Neotropical region
Source: Mem Inst Oswaldo Cruz. 2025 Feb 24;120:e240245. doi: 10.1590/0074-02760240245 (PMC11852318; doi:10.1590/0074-02760240245)
Supplement: Supplementary file 1 [file 1678-8060-mioc-120-e240245-s.pdf]

Supplementary data 1. List of species of tabanids included in this study

Genus	Subgenus	Species	Distribution
"Dasybasis"		*adornata* (Krober)	Peru
		albohirta (Walker)	Argentina
		albosignata (Krober)	Argentina
		albotibialis (Krober)	Peru
		alticola (Enderlein)	Argentina
		andicola (Philippi)	Argentina, Chile
		antilope (Brethes)	Argentina
		antillanca González	Chile
		arauca Coscarón & Philip	Chile
		arica Coscarón & Philip	Chile
		barbata Coscarón & Philip	Chile
		bejeranoi Coscarón & Philip	Argentina
		belenensis Coscarón & Philip	Chile
		boliviame Coscarón & Philip	Bolivia
		bonariensis (Macquart)	Argentina
		brethesi Coscarón & Philip	Argentina
		bruchii (Brethes)	Argentina, Chile
		bulbiscapens Coscarón & Philip	Peru
		bulbula Coscarón & Philip	Bolivia, Chile
		canipilis (Krober)	Argentina
		caprii Coscarón & Philip	Argentina
		chilensis (Macquart)	Argentina, Chile
		chillan Coscarón	Chile
		chubutensis Coscarón	Argentina
		colla Coscarón	Argentina
		collagua González	Chile
		coquimbo Coscarón	Chile
		cumelafquen Coscarón	Argentina
		delpontei Coscarón & Philip	Argentina
		delpontei sepiapes Coscarón & Philip	Chile
		diaguita Coscarón	Argentina
		elquiensis González	Chile
		erynnis (Brethes)	Argentina
		excelsior Fairchild	Ecuador
		fairchildi Coscarón & Philip	Argentina, Peru
		fornesi Coscarón	Argentina
		frequens (Krober)	Bolivia, Peru
		fumifrons Coscarón & Philip	Argentina
		geminata Coscarón & Philip	Peru
		hepperi Coscarón & Philip	Argentina
		hirsuta Coscarón & Philip	Chile
		inata Coscarón & Philip	Chile, Peru
		kroeberi Coscarón & Philip	Chile, Peru

CC BY

		kroeberi picea Coscarón & Philip	Chile
		limbativena (Krober)	Peru
		maletecta (Bigot)	desconocida
		mendozana (Enderlein)	Argentina
		minor (Macquart)	Argentina, Uruguay
		missionum (Macquart)	Argentina, Brazil
		montium (Surcouf)	Bolivia, Colombia, Ecuador, Venezuela
		neogrisescens (Krober)	Peru
		nigra (Enderlein)	Argentina, Chile
		nigrifemur (Krober)	Chile
		nigrifrons (Philippi)	Argentina, Chile
		opaca (Brethes)	Argentina
		ornatissima (Brethes)	Argentina
		pallipes (Krober)	Argentina, Bolivia
		penai Coscarón & Philip	Chile
		pereirai Coscarón & Philip	Argentina, Chile
		pereirai dureti Coscarón & Philip	Argentina, Chile
		persignata (Krober)	Argentina, Bolivia
		pilifer (Krober)	Chile
		poroma Coscarón & Philip	Bolivia
		pruinivitta (Krober)	Argentina, Chile
		punensis (Hine)	Bolivia, Chile, Peru
		schineri (Krober)	Colombia, Ecuador, Venezuela
		schnusei (Krober)	Peru
		senilis (Philippi)	Chile
		shannoni (Krober)	Argentina, Chile
		subtrita Coscarón & Philip	Argentina
		testaceomaculata (Macquart)	Argentina, Chile
		testaceomaculata longifrons (Kröber)	Chile
		testaceomaculata molestissima (Philippi)	Chile
		trigonophora (Macquart)	Argentina, Uruguay
		tritus (Walker)	Argentina, Chile
		vasta Coscarón & Philip	Argentina
		appendiculata Macquart	Australia
		dixoni Ferguson	Australia
		dubiosa Ricardo	Australia
		gentilis Erichson	Australia
		nemopunctata Ricardo	Australia
		trilinealis Ferguson & Henry	Australia
Stenotabanus Lutz	*Stenotabanus* Lutz	*sandyi* Gorayeb	Brazil
		incipiens (Walker)	Guatemala to Bolivia, Argentina, Brazil
		taeniotes (Wiedemann)	Brazil
Scaptiodes Enderlein		*gagatina*	Argentina, Chile

Tabanus Linneaus		*importunus* Wiedemann	Brazil, Bolivia, Panama, Peru
Acellomyia González		*paulseni* (Philippi)	Argentina, Chile
Agelanius Rondani		*meridiana* Rondani	Argentina, Chile
Haematopotina Coscarón & Philip		*argentina* (Brethes)	Argentina
		argentina calchaqui Coscarón & Philip	Argentina, Bolivia
		pechumani Coscarón & Philip	Chile, Peru

Supplementary table II

	Characters and observations	
1	Pollinosity in frons, gena, and subcallus	(0) present
2	Median occipital sclerite	(0) wide, very wide
3	Eyes color	(0) black
4	Ocular ommatrchia	(0) absent
5	Ocular ommatrichia length	(0) microscopic
6	Ocular ommatrichia abundance	(0) scarce
7	Ocular band	(0) present
8	Ocular margin in vertex	(0) present
9	Shape of ocular margin	(0) parallel
10	Shape of frons sides	(0) divergent
11	Frons pilosity color	(0) whitish
12	Frons	(0) with median groove
13	Shape of frons in vertex	(0) convex
14	Band of pruinosity in frons	(0) present
15	Frontal index	(0) up to 2.9
16	Frontal callus presence	(0) present
17	Shape of frontal callus	(0) quadrangular
18	Basal callus	(0) touching the subcallus
19	Basal callus	(0) touching the eyes
20	Surface of callus	(0) rugose
21	Frontal callus	(0) with dorsal-median prolongation
22	Ocelli	(0) present
23	Ocelli state	(0) vestigial
24	Subcallus pilosity	(0) present
25	Longitudinal suture of the subcallus	(0) present
26	Frontoclypeal pilosity	(0) present
27	Pilosity arrangement of the frontoclypeus	(0) lateral
28	Antenna color	(0) bicolered
29	Type of scape	(0) globose, semiglobose
30	Scape pilosity length	(0) short
31	Scape pilosity color	(0) black
32	Pedicel	(0) with dorsal projection
33	Pedicel dorsal projection length	(0) dorsal projection of the pedicel 1/4 to more than the width of the segment
34	Basal flagellomere	(0) with dorsal tooth

CC BY

35	Basal flagellomere	(0) not angular
36	Maxillary palpi length	(0) maxillary palp longer than half the height of the proboscis

37	Maxillary palpi curvature	(0) curved
38	Maxillary palpi pilosity density	(0) sparse
39	Maxillary palpi pilosity color	(2) black
40	Pruinose stripes on mesoscutum	(0) present
41	Shape of prescutellum	(0) triangular
42	Halteres color	(0) dark brown, grayish brown, brown, reddish brown, yellowish brown, light brown
43	Wings	(0) hyaline
44	Basicosta	(0) setulose
45	Subcosta	(0) setulose
46	Vein R1	(0) with several rows of setae
47	Appendix on R4	(0) present
48	Vein CuA2	(0) bare
49	Mid-dorsal abdominal triangles	(0) present
50	Abdominal terga	(0) with median band
51	Abdominal sternum	(0) with median band
52	Shape of female cerci	(0) quadrangular
53	Shape of sternite VIII base	(0) convex
54	Genital fork base	(0) concave or strongly concave
55	Genital fork base	(0) with basal branches
56	Apex of spermathecal ducts	(0) without lateral expansion
57	Lateral projections of spermathecal ducts	(0) presents
58	Spermathecal ducts	(0) short

(1) absent		
(1) narrow		
(1) greenish	(2) blueish	(3) brown
(1) present		
(1) short	(2) long	
(1) abundant		
(1) absent		
(1) absent		
(1) convergent		
(1) convergent	(2) parallel	subparallel
(1) black	(2) brown	(3) yellowish
(1) without median groove		
(1) strait	(2) concave	
(1) absent		
(1) 3 to 5	(2) 5.1 or more	
(1) reduced	(2) absent	
(1) triangular	(2) ovoid	(3) keel-shaped
(1) not touching the subcallus		
(1) not touching the eyes		
(1) smooth		
(1) without dorsal-median prolongation		
(1) absent		
(1) functional	developed	
(1) absent		
(1) absent		
(1) absent		
(1) central and lateral		
(1) unicolored		
(1) not globose		
(1) long		
(1) silver-grayish	(2) whitish	
(1) without dorsal projection		
(1) dorsal projection of the pedicel 1/4 or less of the segment		
(1) without dorsal tooth		
(1) angular		
(1) maxillary palp shorter than half the height of the proboscis	(2) maxillary palp one third of the height of the proboscis	

CC BY

(1) not curved		
(1) dense		
(3) whitish	(4) yellowish	
(1) absent		
(1) bell-shaped		
(1) dark gray, grayish	(2) orange, yellowish, reddish	(3) whitish
(1) with clouds	(2) smoky	
(1) bare		
(1) bare		
(1) with only one row of setae		
(1) absent		
(1) setulose		
(1) absent		
(1) without median band		
(1) without median band		
(1) subcircular	(2) acuminate	(3) subtrapezoidal
(1) concave	(2) straight	(3) sinuous
(1) convex	(2) straight	
(1) without basal branches		
(1) with little expansion	(2) with developed expansion	
(1) absent		
(1) long		

(4) purple

(4) subrectangular

SUPPL data table III

Tabanus	0	1 0	0 ?	?	1	1	0 1	2	1 2	1 2	0	3 1	1
Dasybasis_appendiculata	0	1 0	1 2	1	0	0	0 0	1	1 1	1 0	0	0 0	0
adornata	0	0 0	1 1	1	1	0	0 0	1	1 2	1 0	0	2 0	1
albohirta	0	0 2	0 ?	?	1	0	0 1	1	1 0	1 0	0	2 1	1
albosignata	0	0 3	1 0	0	1	0	0 1	1	1 2	1 0	0	0 0	0
albotibialis	0	0 0	1 2	1	1	0	0 0	?	1 2	1 0	0	0 0	0
alticola	0	0 1	0 0	0	1	0	0 2	1	1 1	1 0	0	0 0	0
andicola	0	0 0	1 2	1	1	0	0 0	1	1 2	1 0	0	0 0	0
antilope	0	0 0	1 1	0	1	0	0 0	1	1 1	1 0	0	0 0	0
arauca	0	0 1	1 0	0	0	0	0 2	1	1 1	1 0	0	1 0	0
arica	0	0 0	1 2	1	1	0	0 0	1	1 1	1 0	0	0 0	0
bejeranoi	0	0 1	0 ?	?	1	0	0 0	1	1 2	1 0	0	0 0	0
belenensis	0	0 0	1 2	1	1	0	0 0	1	1 1	1 0	0	0 0	0
boliviame	0	0 0	1 1	0	0	0	0 0	?	1 0	1 0	0	0 0	0
bonariensis	0	0 2	0 ?	?	0	0	0 0	2	1 2	1 0	0	0 0	0
brethesi	0	0 0	1 2	1	1	0	0 0	1	1 2	1 0	1	2 0	1
bruchii	0	0 0	1 1	0	1	0	0 1	1	1 2	1 0	0	0 0	0
bulbula	0	0 0	1 2	1	1	0	0 2	1	1 2	1 0	0	0 0	0
canipilis	0	0 1	1 1	0	1	0	0 2	1	1 1	1 0	0	0 0	0
caprii	0	0 1	0 ?	?	1	0	0 2	1	1 1	1 0	0	0 0	0
chilensis	0	0 0	1 2	1	1	1	0 0	1	1 2	1 0	0	0 0	1
chillan	0	0 0	1 2	1	1	0	0 0	1	1 1	1 0	0	2 0	1
chubutensis	0	0 2	1 0	0	1	0	0 0	1	1 2	1 0	0	0 0	0
colla	0	0 1	1 2	1	1	0	0 0	1	1 2	1 0	0	0 0	0
coquimbo	0	0 0	1 2	1	1	0	0 0	1	1 2	1 0	0	2 0	1
cumelafquen	0	0 0	1 1	1	1	0	0 2	1	1 1	1 0	0	0 0	0
delpontei	0	0 0	1 1	1	1	0	0 0	1	1 2	1 0	0	0 0	0
delpontei_sepiapes	0	0 0	1 2	1	1	0	0 0	1	1 2	1 0	0	0 0	1
diaguita	0	0 0	1 1	0	1	0	0 0	1	1 2	1 0	0	1 0	0
elquiensis	0	0 0	1 1	0	1	0	0 0	1	1 1	1 0	0	0 0	0
erynnis	0	0 0	0 ?	?	1	0	0 2	1	1 1	1 0	0	0 0	0
excelsior	0	1 0	1 1	0	0	1	0 2	1	1 1	1 1	0	2 0	1
fairchildi	0	0 1	1 2	1	1	0	0 1	1	1 2	1 0	0	2 0	1
fornesi	0	0 0	1 1	0	0	0	0 2	1	1 2	1 0	0	2 0	0
frequens	0	0 ?	1 1	1	1	0	0 2	1	1 2	1 0	0	0 0	0
fumifrons	0	0 0	1 1	0	1	0	0 0	1	1 2	1 0	0	0 0	0
geminata	0	0 0	1 1	0	1	0	0 0	1	1 ?	1 0	0	1 ?	?
hepperi	0	0 ?	1 1	0	1	0	0 0	1	1 1	1 0	0	0 0	0
hirsuta	0	0 0	1 2	1	1	0	0 0	1	1 2	1 0	0	0 0	0
inata	0	0 0	1 2	1	1	0	0 0	1	1 1	1 0	0	1 0	0
kroeberi	0	0 0	1 1	0	0	0	0 0	1	1 1	1 0	0	0 0	0
kroeberi_picea	0	0 0	1 1	0	1	0	0 0	1	1 1	1 0	0	0 0	0

limbativena	0	0 0	1 2	1	1	0	0 2 1	1 2	1 0 0	0 0 0

mendozana	0	0	1	1	0	0	0	0	0	0	1	1	2	1	0	0	0	0	0
minor	0	0	1	1	0	0	0	0	0	0	1	1	1	1	0	0	0	0	0
missionum	0	0	0	0	?	?	0	0	0	2	1	1	1	1	0	0	0	0	0
montium	0	0	0	1	2	1	1	0	0	0	1	1	2	1	0	0	0	0	0
neogrisescens	0	?	?	1	1	0	1	0	?	2	1	1	1	1	0	0	0	0	0
nigra	0	0	0	1	1	1	1	0	0	0	1	1	1	1	0	0	0	0	0
nigrifemur	0	0	?	?	?	?	?	0	?	0	?	1	1	1	0	0	0	0	0
nigrifrons	0	0	0	1	1	0	1	0	0	0	1	1	2	1	0	0	0	0	0
antillanca	0	0	0	1	2	1	1	0	0	2	1	1	1	1	0	0	2	1	1
opaca	0	0	0	1	2	1	1	0	0	0	1	1	2	1	0	2	2	0	1
ornatissima	0	?	1	1	0	0	0	0	?	2	?	1	2	?	0	0	2	0	1
pallipes	0	0	1	1	1	1	1	0	0	2	2	1	0	1	0	0	0	0	0
penai	0	0	0	1	2	1	1	0	0	0	1	1	2	1	0	0	0	0	0
pereirai	0	0	0	1	2	1	1	0	0	0	1	1	2	1	0	0	0	0	0
pereirai_dureti	0	0	0	1	2	1	1	0	0	0	1	1	2	1	0	0	0	0	0
persignata	0	0	0	0	?	?	1	0	0	1	1	1	1	1	0	0	2	0	1
pilifer	0	0	?	?	?	?	?	0	?	2	?	1	1	1	0	0	0	0	0
poroma	0	0	0	1	1	0	1	0	0	2	1	1	2	1	0	0	0	0	0
pruinivitta	0	0	0	1	2	1	1	0	0	0	1	1	1	1	0	0	0	0	0
punensis	0	0	1	1	?	1	1	0	0	2	1	1	2	1	0	0	0	0	0
schineri	0	0	?	1	2	1	1	0	0	2	1	1	0	1	0	0	0	0	0
schnusei	0	0	0	1	0	0	0	0	?	2	1	1	1	1	0	0	0	0	0
senilis	0	0	0	1	2	1	1	0	0	2	1	1	2	1	0	0	0	0	0
shannoni	0	0	0	1	0	0	1	0	0	2	1	1	1	1	0	0	2	0	0
subtrita	0	0	3	1	0	0	0	0	0	2	1	1	2	1	0	0	0	0	0
testaceomaculata	0	0	0	1	1	0	1	0	0	2	1	1	1	1	0	0	0	0	1
testaceomaculata_longifrons	0	0	0	1	1	0	1	0	0	2	1	1	0	1	0	0	0	0	0
testaceomaculata_molestissima	0	0	0	1	1	0	1	0	0	2	1	1	1	1	0	0	0	0	0
trigonophora	0	0	?	1	0	0	1	0	0	2	1	1	1	1	0	0	0	0	0
tritus	0	0	0	1	0	0	0	0	0	0	1	1	1	1	0	0	0	0	0
vasta	0	0	1	1	2	1	0	0	0	2	1	1	1	1	0	0	0	0	0
collagua	0	0	0	1	2	1	1	0	0	2	1	1	1	1	0	0	0	0	0
Agelanius	0	1	3	1	2	1	1	0	0	2	1	1	1	1	1	0	3	1	1
Acellomyia	0	0	3	1	1	0	1	0	0	0	1	1	1	1	0	0	2	1	1
Haematopotina	0	0	3	1	0	0	1	0	1	2	0	1	1	0	0	0	0	0	0
Nubiloides	0	0	3	1	1	1	1	1	0	2	1	1	1	1	0	0	3	1	1
Haematopotina_argentina	0	0	0	1	0	0	1	0	1	2	0	1	1	0	0	0	0	0	0
Haematopotina_pechumani	0	0	3	1	2	1	1	0	1	0	0	1	2	0	0	0	0	0	0
Scaptiodes_gagatina	1	0	1	1	2	1	0	0	0	0	1	0	2	1	0	0	0	0	1
Dicladocera	0	0	3	1	1	1	1	1	0	2	1	1	1	1	1	0	3	0	1
Stenotabanus_sandyi	0	0	3	0	?	?	0	0	0	1	1	1	1	1	1	0	0	0	0
Stenotabanus_incipiens	0	1	0	1	1	0	0	0	?	1	0	1	2	1	1	0	0	0	0
nemopunctata	0	1	1	0	?	?	1	1	0	2	2	1	1	1	1	2	3	1	1

trilinealis	0	1	0	0	?	?	1	0	0	2	2	1	2	1	1	0	0	0	1
dubiosa	0	1	0	1	0	0	1	0	0	0	1	1	0	1	0	0	0	0	?
dixoni	0	1	0	1	0	0	1	0	0	0	2	1	0	1	0	1	3	?	1
gentilis	0	1	0	1	2	1	1	0	0	0	1	1	1	1	0	0	0	?	0
Stenotabanus_taeniotes	0	0	4	0	?	?	0	0	0	1	3	1	1	0	1	0	3	0	0

1	0	1	?	1	00	1	0	1	02	0	0	0	1	0	0	1	01	0	0	2	0	0	0
1	0	1	?	0	00	1	0	1	10	1	?	1	0	0	1	1	01	0	0	2	0	0	0
0	0	0	0	0	0?	?	?	1	10	?	?	?	?	1	0	1	00	?	0	0	1	?	0
1	1	1	?	1	00	1	0	1	02	0	1	1	1	1	0	1	10	0	3	0	1	0	0
1	1	0	0	1	00	0	1	1	00	1	?	1	1	1	0	1	10	0	1	0	1	0	0
0	0	1	?	0	0?	?	0	1	12	0	1	1	1	1	0	1	10	0	2	0	1	0	?
1	1	0	1	0	00	0	0	1	02	0	0	1	1	0	1	1	10	0	0	1	1	0	0
0	1	1	?	0	00	1	0	1	10	0	0	1	1	1	0	1	00	0	1	0	1	0	0
1	1	0	1	0	00	0	0	1	00	1	?	1	1	1	1	1	00	0	0	1	1	0	0
1	1	1	?	0	00	1	0	1	00	0	1	1	1	0	1	1	00	0	0	1	1	0	0
1	1	0	1	0	00	1	1	1	10	0	1	1	1	0	1	1	00	0	0	0	1	0	0
0	1	0	1	1	00	1	0	1	02	?	?	1	1	0	1	0	10	0	0	0	1	0	0
0	0	1	?	0	00	1	0	1	10	0	0	1	1	0	1	1	00	0	0	2	1	0	0
1	1	1	?	0	0?	?	?	1	02	?	?	?	?	0	1	?	10	0	2	1	1	0	0
1	0	1	?	1	0?	?	0	1	00	0	0	1	1	0	1	0	10	0	?	1	1	0	0
0	1	0	0	0	0?	?	1	1	10	0	1	1	0	1	0	1	00	0	0	0	1	1	0
1	0	1	?	1	00	0	0	1	00	0	1	1	1	1	0	1	00	0	0	0	1	0	0
1	1	1	?	0	00	1	1	0	10	0	0	1	1	2	1	1	10	0	0	1	1	0	0
0	1	0	1	0	00	1	1	1	00	0	1	1	1	0	1	1	00	0	0	0	1	0	0
1	0	1	?	1	0?	?	0	1	12	0	0	1	1	0	1	1	00	0	?	0	1	0	0
1	1	1	?	0	00	1	0	1	10	0	0	1	1	1	0	1	10	0	0	0	1	0	0
0	0	0	0	0	00	1	1	1	10	0	1	1	1	0	1	1	00	0	0	0	1	0	0
1	1	1	?	0	00	1	0	1	02	0	0	1	0	0	1	1	00	0	0	1	1	0	0
0	0	1	?	0	0?	?	1	1	11	1	?	1	1	1	0	1	00	?	?	1	1	0	0
0	0	0	0	0	0?	?	1	1	10	0	0	1	1	1	0	1	00	?	?	2	1	0	0
0	1	1	?	0	0?	?	1	1	00	0	0	1	1	0	1	1	00	?	0	0	1	0	0
0	1	0	0	0	00	1	0	1	10	1	?	1	1	1	0	1	00	0	0	0	1	0	0
0	1	0	0	0	00	1	0	1	10	1	?	1	1	1	0	1	00	0	0	0	1	0	0
1	0	0	0	0	0?	?	1	1	10	0	0	1	1	1	0	0	00	?	?	0	1	0	0
1	1	1	?	0	00	0	0	1	00	0	1	1	1	1	0	0	00	0	0	0	1	0	0
1	0	1	?	1	00	1	0	1	00	1	?	1	1	0	1	1	00	0	0	1	1	0	0
0	0	0	0	0	00	1	0	1	10	0	1	1	1	0	1	1	00	0	0	1	1	0	0
0	1	1	?	0	0?	?	?	0	11	0	0	1	?	2	1	1	00	0	0	1	1	0	0
1	0	1	?	0	00	0	1	1	00	0	1	1	1	1	0	1	00	0	0	2	1	0	0
1	0	0	0	0	0?	?	1	1	10	0	1	1	0	1	0	1	00	0	?	0	1	0	0
0	0	1	?	0	0?	?	?	1	00	0	1	1	0	1	0	1	00	0	0	0	1	0	0
?	0	0	1	?	0?	?	0	1	11	0	1	1	1	1	0	1	10	0	0	0	1	1	0
1	1	1	?	0	0?	?	0	1	0?	0	1	1	1	1	1	0	00	0	0	0	1	0	?
0	1	0	0	0	00	1	1	0	12	0	0	1	0	2	1	1	0 0&1	0	0	0	1	0	0
0	0	0	1	0	00	1	0	1	10	0	0	1	0	1	0	1	00	0	0	0	1	0	0
0	0	0	1	0	00	1	0	1	10	0	0	1	1	1	0	1	10	0	0	1	1	0	0
1	0	0	1	0	00	1	0	1	12	0	0	1	1	1	0	1	10	0	0	1	1	0	0
1	1	1	?	0	0?	?	0	0	1?	0	0	1	0	2	1	1	00	0	2	1	1	0	0

1	1	0	1	0	0	0	1	0	1	0	0	0	0	1	1	0	1	1	0	0	0	0	1	1	0	0
1	0	1	?	1	0	0	1	0	1	0	0	1	?	1	1	0	1	1	0	0	0	2	0	1	0	0
1	0	0	0	1	?	0	1	0	1	0	2	1	?	1	0	0	1	1	1	1	0	0	0	1	0	0
1	1	1	?	0	0	0	1	0	1	1	0	0	0	1	1	1	0	1	0	0	0	0	1	1	0	0
1	1	1	?	0	0	?	?	0	0	1	0	0	0	1	0	2	1	1	0	0	?	0	1	?	?	?
1	1	0	0	0	0	0	1	1	1	0	1	0	0	1	1	1	0	1	0	0	0	0	1	1	0	0
1	1	0	0	1	0	?	?	0	1	?	?	?	?	1	0	1	0	1	0	0	?	?	0	1	?	?
1	1	0	0	0	0	0	0	0	1	1	2	1	?	1	1	1	0	1	0	0	0	0	0	1	0	0
1	1	1	?	0	0	0	0	0	1	0	2	0	0	1	0	0	1	1	1	0	0	0	1	1	0	0
0	1	0	1	0	0	0	1	1	1	1	0	1	?	1	1	0	1	1	0	1	0	0	0	1	0	0
1	1	1	?	0	0	?	?	0	1	0	0	0	0	1	1	0	1	1	0	0	?	?	1	1	?	?
0	1	1	?	0	0	0	0	0	1	1	0	1	?	1	1	1	0	1	0	0	0	0	0	1	0	0
0	0	1	?	0	0	?	?	0	0	1	0	0	0	1	1	1	0	1	2	1	?	0	1	1	0	0
0	1	0	1	0	0	0	1	0	1	1	0	0	1	1	1	0	1	1	2	0	0	0	0	1	0	0
0	1	0	0	0	0	0	1	0	1	1	0	0	1	1	1	0	1	1	0	0	0	0	0	1	0	0
1	1	1	?	0	0	0	1	0	1	0	0	0	1	1	1	1	0	1	0	0	0	2	0	1	0	0
0	1	1	?	0	0	?	?	0	1	1	?	0	0	1	1	1	0	1	2	1	?	?	0	1	?	?
0	1	1	?	0	0	?	?	1	0	1	2	0	0	1	0	2	1	1	1	0	?	0	1	1	1	0
1	1	0	0	0	0	0	0	0	1	1	0	0	1	1	1	1	0	1	0	0	0	0	0	1	0	0
0	0	0	0	0	0	?	?	1	1	1	0	0	0	1	0	0	1	1	0	0	?	?	0	1	0	0
0	1	1	?	0	0	?	?	1	0	1	0	1	?	1	1	0	1	1	0	0	?	0	1	0&1	0	?
1	1	0	0	0	0	?	?	0	1	0	?	0	1	1	1	0	1	1	0	0	?	0	0	1	?	?
0	1	0	0	0	0	0	0	0	1	1	2	0	1	1	1	0	1	1	1	0	0	0	0	1	0	0
0	1	0	1	0	0	0	0	0	1	0	0	0	1	1	1	0	1	1	0	0	0	0	1	1	0	0
1	0	0	1	0	0	0	0	1	1	0	0	0	0	1	1	0	1	0	0	0	0	0	1	1	0	0
0	1	0	0	1	0	0	1	0	1	0	0	0	1	1	1	1	0	1	0	0	0	0	0	1	0	0
0	0	0	0	0	0	0	1	0	1	0	0	0	1	1	1	1	0	1	0	0	0	0	0	1	0	0
0	1	0	0	0	0	0	1	0	1	0	0	0	1	1	1	1	0	1	0	0	0	0	0	1	0	0
1	1	1	?	0	0	?	?	0	1	0	0	0	1	1	1	0	1	1	0	1	0	0	0	1	0	0
1	0	1	?	0	0	0	0	0	1	0	0	0	1	1	1	0	1	1	2	0	0	0	1	1	0	0
0	0	1	?	0	0	0	0	0	0	1	0	0	1	1	1	0	1	1	0	0	0	2	1	1	0	0
0	1	1	?	0	0	0	1	0	0	1	2	0	0	1	0	2	1	1	0	1	0	0	0	1	0	0
1	0	0	1	1	0	0	0	0	1	0	0	0	0	1	1	0	1	1	0	0	0	0	1	1	0	0
1	0	1	?	0	0	0	0	0	1	0	0	0	0	1	0	0	1	1	0	0	0	0	1	1	0	0
1	1	1	?	1	0	0	0	0	0	1	2	0	1	1	0	2	0	1	0	0	0	0	1	1	1	1
1	0	0	0	0	0	0	0	1	1	0	0	0	0	0	1	0	1	1	0	0	1	0	2	1	0	0
1	1	1	?	1	0	0	0	0	1	0	2	0	1	1	0	1	0	1	1	0	0	0	1	0	1	1
1	1	1	?	0	0	0	0	0	0	1	2	0	1	1	0	1	0	1	0	0	0	0	1	0	1	1
0	1	0	1	1	0	1	?	1	1	1	0	1	?	1	1	1	0	1	0	1	0	0	2	1	0	0
0	0	1	?	0	0	0	0	0	1	0	0	0	0	0	0	0	1	1	0	1	0	0	2	1	1	0
1	0	0	0	1	1	1	?	0	1	0	0	0	1	1	1	1	0	1	0	1	?	0	2	1	0	0
1	0	0	0	0	0	0	1	1	1	0	1	0	1	1	0	0	1	1	0	1	1	0	0	1	0	0
0	0	1	?	1	0	0	1	1	1	0	1	0	1	1	1	0	1	1	2	1	0	0	0	0	0	0

1	0	0	0	1	0	0	1	0	1	0	0	0	1	1	1	0	1	1	0	0	1	0	0	0	0	0
1	0	1	?	1	0	0	0	0	1	0	1	0	1	1	1	0	1	1	0	0	0	0	0	0	0	0
0	0	1	?	1	0	0	0	0	1	0	1	0	1	1	1	0	1	1	1	0	0	0	0	0	0	0
0	0	1	?	1	0	0	1	0	1	0	1	0	1	1	1	0	1	1	0	0	0	0	1	0	0	0
1	0	0	0	0	0	0	1	0	1	0	0	1	1	1	1	0	1	0	2	1	1	0	0	1	0	0

1	1	0	0	1	0	1	0	1	2	0	1		
0	0	0	1	1	1	2	0	1	0	1	1		
0	0	0	1	1	?	?	?	?	?	?	?		
0	0	1	0	1	1	1	0	0	2	0	0		
0	0	0	1	1	1	2	2	1	2	0	0		
0	0	1	1	?	2	0	2	1	2	0	0		
0	0	1	0	1	0	1	0	1	2	0	0		
1	0	1	1	0	1	2	0	0	2	0	0		
0	0	1	0	1	0	0	0	1	2	0	0		
0	0	0	1	1	0	0	0	1	2	0	0		
0	0	0	1	1	3	0	0	1	2	0	0		
0	0	0	1	1	0	2	2	1	2	0	0		
0	0	1	0	1	1	0	0	1	2	0	0		
0	0	1	0	1	0	3	0	?	2	0	0		
0	0	1	0	1	0	0	0	1	2	0	0		
1	0	1	1	1	0	0	2	1	2	0	0		
0	0	0	1	0	1	1	0	1	2	0	0		
0	0	1	1	?	?	?	?	?	?	?	?		
0	0	0	1	1	0	0	0	1	2	0	0		
0	0	0	0	?	0	2	0	1	2	0	0		
1	0	0	1	0	1	0	0	1	2	0	0		
1	0	1	1	1	2	1	2	1	2	0	0		
0	0	0	1	1	2	0	2	1	2	0	0		
1	0	1	1	1	0	2	2	1	2	0	0		
1	0	1	1	1	1	0	0	1	2	0	0		
0	0	0	1	1	1	0	0	1	2	0	0		
1	0	0	1	1	1	1	0	1	2	0	0		
1	0	0	1	1	1	1	0	1	2	0	0		
0	0	0	1	?	0	0	0	1	2	0	0		
1	0	0	1	1	1	2	0	1	2	0	0		
0	0	1	0	0	0	2	0	1	2	0	0		
0	1	1	1	1	?	?	?	?	?	?	?		
0	0	0	1	1	1	2	0	1	2	0	0		
0&1	0	1	1	1	0	0	1	1	2	0	0		
1	0	0	1	1	1	0	0	0	2	0	0		
1	0	0	1	?	2	1	?	?	2	0	0		
0	0	0	1	?	?	?	?	?	?	?	?		
0	0	0	1	1	1	0	2	1	2	0	0		
1	0	1	0	1	0	0	0	0	2	0	0		
1	0	0	1	0	3	0	0	1	2	0	0		
0	0	0	1	1	4	2	2	1	2	0	0		
0	0	0	1	1	0	2	0	1	2	0	0		
0	0	0	1	1	?	?	?	?	?	?	?		

0	0	0	1	0	1	1	0	1	2	0	0
0	0	1	0	1	0	2	0	1	2	0	0
0	0	1	0	0	0	2	0	1	2	0	0
1	0	0	1	0	1	2	0	0	2	0	0
0	?	1	1	1	?	?	?	?	?	?	?
0	0	1	1	1	0	0	2	1	2	0	0
1	0	1	0	?	?	?	?	?	?	?	?
1	0	1	1	1	2	1	0	0	2	0	0
1	0	0	1	0	?	2	0	1	2	0	0
1	0	1	1	?	2	2	0	1	2	0	0
0	?	0	0	1	1	1	0	1	2	0	0
1	0	0	1	1	1	2	0	1	2	0	0
1	0	0	1	0	1	0	0	1	2	0	0
1	0	0	1	0	2	3	0	1	2	0	0
1	0	0	1	0	2	3	0	1	2	0	0
1	0	0	0	1	0	2	0	0	2	0	0
1	0	1	1	1	?	?	?	?	?	?	?
0	0	1	1	1	1	1	0	1	2	0	0
0	0	0	1	1	1	2	0	?	2	0	0
0&1	0	0	1	0	1	0	0	1	2	0	0
?	0	0	1	0	1	1	0	0	2	0	0
0	?	1	1	1	?	?	?	1	?	?	?
1	0	1	1	1	0	3	0	1	2	0	0
0	0	0	1	0	2	0	0	1	2	0	0
0	0	0	1	0	1	2	0	1	2	0	0
1	0	1	0	0	1	2	0	1	2	0	0
1	0	1	1	0	1	2	0	1	2	0	0
1	0	1	1	0	0	3	0	1	2	0	0
0&1	0	0	1	0	1	2	0	1	2	0	0
0	0	0	1	1	0	2	0	?	2	0	0
0	0	1	0	1	4	2	0	1	2	0	0
1	0	1	1	1	1	3	0	1	2	0	0
0	0	0	1	1	1	2	0	1	2	0	1
0	0	0	1	1	1	2	0	1	1	0	0
1	0	0	0	1	0	0	0	0	1	0	0
1	0	0&1	0	1	1	2	0	1	2	0	1
1	0	0	0	1	0	0	0	0	1	0	0
1	0	0	1	1	0	0	0	0	1	0	0
1	1	1	1	1	1	2	0	1	1	0	1
1	0	1	1	1	0	1	0	1	2	0	1
1	0	1	0	1	1	0	0	0	2	0	1
0	0	0	0	1	1	0	0	0	0	1	1
0	0	1	1	1	1	0	1	1	0	1	1

0	0	0	0	0	0	0	2	1	0	1	1
0	0	0	1	1	1	0	1	1	0	1	1
0	0	0	1	1	1	0	0	1	0	1	1
0	0	0	1	1	1	0	1	1	0	1	1
1	0	1	0	1	1	0	0	0	2	0	1

Supplementary Table IV
A TPS file for _*Dasybasis*_ wing landmarks (30 Landmarks)

```
LM=30
 4.088370 8.414550
 3.232770 9.425710
 3.932800 10.009070
 4.477280 10.553540
 4.438380 11.020230
 6.460710 10.242420
 12.177670 10.981340
 15.211160 10.942450
 19.411370 10.086850
 20.266970 9.347930
 17.427940 5.769970
 16.922360 5.458840
 15.677850 4.992150
 13.888870 4.525460
 12.527690 4.369890
 10.077560 4.292110
 9.688650 4.797690
 8.366360 7.442270
 9.260850 7.675620
 9.494200 7.908960
 8.794160 8.881240
 12.255450 6.625560
 9.999780 8.920130
 9.260850 9.970180
 10.116450 9.347930
 13.344390 6.936690
 13.577740 7.481160
 15.522280 8.025640
 15.794520 8.531220
 NA  NA
ID=Tabanus


LM=30
 3.732610 7.236300
 3.265920 7.936330
 3.810390 8.286350
 3.849280 8.675260
 3.927060 9.025280
 5.404920 8.441910
 9.410680 8.908600
 9.760690 8.869710
 14.077580 8.403020
 15.049850 7.858550
 13.844230 5.252860
 12.910850 4.747280
 12.094150 4.475040
 11.082980 4.125020
```

```
 9.877370 3.969460
 7.971710 3.891680
 7.660590 4.436150
 7.116110 6.186240
 7.855040 6.419590
 8.010600 6.536260
 7.349460 7.391860
 9.799580 5.719550
 8.088390 7.469640
 7.543910 8.208570
 8.049490 7.702990
 10.577400 5.952900
 10.616290 6.302910
 12.171930 6.730710
 12.405270 7.080730
 12.094150 7.236300
ID=Dasybasis_appendiculata


LM=30
 2.813460 7.207530
 2.525580 7.840870
 3.101340 8.128750
 3.130130 8.531790
 3.158920 8.819670
 4.454400 8.330270
 8.081720 8.531790
 9.434770 8.589370
 12.428760 8.042390
 12.975740 7.610560
 11.392380 5.509020
 10.931770 5.307500
 10.068120 4.990830
 9.031740 4.846890
 8.139300 4.674160
 6.412000 4.674160
 6.181690 5.105980
 5.577140 6.459030
 6.296850 6.631760
 6.354420 6.746920
 5.980180 7.380260
 8.081720 5.969630
 6.613520 7.437830
 6.325640 8.013600
 6.728670 7.754510
 8.772640 6.257510
 8.887800 6.660550
 10.442360 6.746920
 10.643880 6.919650
 NA  NA
ID=albohirta


LM=30
 6.161910 6.611090
 5.825800 7.130540
 6.345250 7.466650
```

```
 7.628580 7.619420
 6.436910 7.711090
 6.406360 7.955540
 9.920250 7.863870
 10.592470 7.924980
 13.220250 7.558310
 13.861910 7.038870
 12.792470 5.602760
 11.998030 5.297200
 11.295250 5.144420
 10.714690 5.022200
 10.134140 4.899980
 8.850800 4.838870
 8.667470 5.113870
 8.300800 6.091650
 8.728580 6.244420
 8.820250 6.427760
 8.606360 6.855530
 10.073030 5.908310
 9.034140 6.916650
 8.820250 7.405540
 9.095250 7.130540
 10.500800 6.122200
 10.592470 6.366650
 11.906360 6.427760
 12.028580 6.641650
 11.875800 6.702760
ID=albosignata


LM=30
 3.166270 5.052760
 2.799600 5.663870
 3.380160 6.122200
 3.807940 6.488870
 3.807940 6.733310
 5.091270 6.305530
 9.185720 6.549980
 10.255160 6.488870
 13.891270 5.816650
 14.655160 5.266650
 12.821830 2.822200
 12.057940 2.486090
 11.171830 2.241650
 9.949600 1.997200
 9.032940 1.874980
 6.863490 1.905530
 6.771830 2.272200
 5.977380 4.288870
 6.863490 4.258310
 6.955160 4.441650
 6.588490 5.144420
 8.910720 3.555530
 7.382940 5.144420
 7.046830 5.938870
 7.444050 5.480530
 9.705160 3.830530
```

```
 9.857940 4.136090
 11.477380 4.288870
 11.630160 4.686090
 11.355160 4.869420
ID=alticola


LM=30
 3.607660 3.877870
 2.989450 4.518980
 3.561870 5.022710
 3.859530 5.457750
 3.790840 5.915680
 5.828650 5.251680
 10.247730 5.938580
 11.209400 5.869890
 15.514000 5.320370
 16.452770 4.725050
 14.987370 2.504060
 14.140190 2.000330
 13.018250 1.496600
 11.690230 1.130250
 10.568290 0.855490
 8.255710 0.786800
 7.935160 1.153150
 7.294050 3.076480
 8.095430 3.236760
 8.164120 3.488620
 7.545910 4.221320
 10.430910 2.504060
 8.530470 4.312910
 8.141230 5.091400
 8.644960 4.633460
 11.415470 2.962000
 11.529950 3.442830
 13.315900 3.671800
 13.544870 3.923660
 NA  NA
ID=andicola


LM=30
 3.694010 5.509020
 3.233400 6.113570
 3.694010 6.487820
 3.953100 6.833280
 3.895530 7.121160
 5.075850 6.660550
 8.444080 6.919650
 8.962270 6.890860
 12.388080 6.430240
 13.021420 5.998420
 11.639580 4.012030
 11.035030 3.666560
 10.459260 3.407470
 9.538040 3.119590
 8.645600 3.004430
```

```
 7.148610 2.975650
 6.918300 3.205950
 6.256170 4.818100
 6.918300 4.875670
 6.947090 5.048400
 6.601630 5.652960
 8.588020 4.328700
 7.148610 5.710540
 6.803150 6.401460
 7.206180 5.998420
 9.106210 4.587790
 9.192580 4.933250
 10.516840 5.221130
 10.631990 5.451440
 10.344110 5.624170
ID=antilope


LM=30
 3.786290 3.210150
 3.103940 3.873550
 3.767340 4.385310
 3.805250 4.745440
 3.748380 5.029760
 5.662760 4.650670
 9.301980 5.238250
 10.041200 5.351980
 14.343810 5.314070
 15.215710 4.916030
 13.547730 2.357210
 12.789560 1.997080
 11.936620 1.655900
 10.894140 1.257860
 9.908520 1.068320
 8.354270 0.840870
 8.051000 1.144130
 7.008520 2.906880
 7.652960 3.020610
 7.728780 3.172240
 7.235970 3.759820
 9.643160 2.527790
 8.126820 3.873550
 7.671910 4.574850
 8.259500 4.214720
 10.401330 2.850020
 10.496100 3.267010
 12.315710 3.627140
 12.486290 3.949360
 11.500670 4.214720
ID=arauca


LM=30
 4.211950 5.537480
 3.744450 6.362480
 4.294450 6.664980
 4.459450 7.077480
```

```
 4.431950 7.434980
 6.026950 6.884980
 9.684450 7.297480
 10.729450 7.297480
 13.946950 6.719980
 14.799450 6.279980
 13.259450 4.327480
 12.461950 3.914980
 11.664450 3.584980
 10.811950 3.227480
 9.904450 3.062480
 8.061950 2.952480
 7.924450 3.117480
 7.264450 4.712480
 7.896950 4.959980
 8.034450 5.207480
 7.566950 5.867480
 9.656950 4.547480
 8.144450 5.977480
 7.786950 6.719980
 8.281950 6.252480
 10.371950 4.904980
 10.481950 5.234980
 12.021950 5.454980
 12.104450 5.647480
 11.856950 5.812480
ID=arica


LM=30
 3.757860 6.084780
 3.354820 6.862070
 3.959380 7.207530
 3.930590 7.639350
 4.016950 7.927240
 5.801830 7.437830
 9.170060 7.984810
 10.148870 7.984810
 13.574670 7.236320
 14.467110 6.804490
 13.229220 4.875670
 12.279200 4.415060
 11.357970 3.896870
 10.350380 3.724140
 9.659460 3.580200
 7.845800 3.465050
 7.586710 3.868080
 6.867000 5.422650
 7.500340 5.509020
 7.701860 5.796900
 6.982150 6.574180
 9.342790 5.221130
 7.759440 6.631760
 7.471550 7.265110
 7.932170 6.862070
 9.976140 5.422650
 10.062500 5.796900
```

```
 11.789800 5.998420
 12.020110 6.343880
 11.761010 6.459030
ID=belenensis


LM=30
 3.904540 3.537760
 3.376090 4.087350
 3.967960 4.700360
 4.306170 5.080850
 4.200480 5.419060
 6.081780 4.975150
 10.689910 5.440190
 11.641130 5.503610
 15.974470 4.827190
 16.883410 4.171900
 15.107800 1.719870
 14.262270 1.297100
 13.247640 0.980030
 11.873650 0.747510
 11.028120 0.578400
 8.872030 0.747510
 8.491540 1.085720
 7.709420 2.861330
 8.385850 2.903610
 8.512680 3.114990
 7.963080 3.875970
 10.732190 2.290600
 8.829750 3.918240
 8.428120 4.679220
 8.977720 4.277590
 11.619990 2.480840
 11.767960 2.882470
 13.480160 3.262960
 13.628120 3.601170
 13.289910 3.812550
ID=bonariensis


LM=30
 3.144810 5.930510
 2.634940 6.440380
 3.258120 6.865280
 3.428080 7.261850
 3.456400 7.516790
 5.042680 6.950260
 8.583490 7.233520
 9.914830 7.233520
 12.832450 6.610340
 13.738900 6.128790
 12.322580 4.032640
 11.416130 3.664390
 10.509690 3.409450
 9.631570 3.267820
 8.696790 3.154520
 6.883900 3.154520
```

```
 6.685620 3.381130
 6.147410 5.109040
 6.713940 5.165690
 6.912230 5.335650
 6.459010 6.015490
 8.668470 4.457530
 7.195490 5.987160
 6.883900 6.667000
 7.308800 6.270430
 9.404960 4.655820
 9.603240 5.024060
 10.962910 5.307330
 11.076220 5.505610
 NA  NA
ID=bruchii


LM=30
 3.733570 6.491870
 3.431450 7.096120
 3.925830 7.563030
 4.173030 7.947550
 4.063160 8.249680
 5.820970 7.727830
 9.418970 7.920090
 10.462670 7.892620
 13.676150 7.123580
 14.362800 6.546800
 12.824720 4.679140
 12.055680 4.322080
 11.204240 4.074890
 10.105610 3.855170
 9.364040 3.772770
 7.413980 3.882630
 7.331580 4.019960
 6.727330 5.750300
 7.304110 5.832700
 7.496370 6.024960
 7.056920 6.711600
 9.061920 5.283380
 7.743570 6.766530
 7.413980 7.480640
 7.825960 7.041180
 9.803490 5.585500
 9.940820 5.832700
 11.616230 5.860160
 11.835950 6.024960
 11.616230 6.244680
ID=bulbula


LM=30
 3.172600 5.143010
 2.602200 5.713400
 3.276310 6.206020
 3.535580 6.569000
 3.535580 6.906060
```

```
 5.013430 6.309730
 8.695100 6.491220
 10.302590 6.517150
 13.284220 5.868970
 14.036110 5.350420
 12.247130 3.276240
 11.573030 2.965120
 10.717430 2.679920
 9.628480 2.472500
 8.695100 2.316940
 6.906120 2.420640
 6.828340 2.472500
 6.050520 4.442970
 6.724630 4.468900
 6.828340 4.598530
 6.413500 5.272640
 8.643250 3.768860
 7.113540 5.350420
 6.932050 6.050460
 7.295030 5.583770
 9.291430 4.002210
 9.421060 4.365190
 11.132260 4.520750
 11.287830 4.754100
 10.924840 5.013370
ID=canipilis


LM=30
 4.192490 2.967800
 3.731440 3.482050
 4.281150 3.907630
 4.316620 4.173620
 4.334350 4.528280
 5.894830 4.138160
 8.927120 4.616940
 10.877720 4.705610
 12.899250 4.492810
 13.750420 4.155890
 12.615530 1.992500
 11.799820 1.549180
 11.037320 1.318660
 10.274810 0.964000
 9.459110 0.751210
 7.774500 0.591620
 7.614900 0.857610
 6.887860 2.400350
 7.419840 2.471280
 7.543970 2.666340
 7.118390 3.286990
 9.175380 2.098900
 7.756770 3.411120
 7.384380 3.978560
 7.845430 3.641640
 9.707360 2.382620
 9.796030 2.684080
 11.161450 3.074200
```

```
 11.232380 3.251520
 NA  NA
ID=caprii


LM=30
 2.568420 2.939380
 2.101760 3.456050
 2.568420 3.872720
 2.718420 4.189380
 2.601760 4.456050
 4.218420 4.072720
 7.568420 4.306050
 8.185090 4.306050
 11.818420 3.706050
 12.668420 3.189380
 11.085090 1.439380
 10.385090 1.122720
 9.568420 0.872720
 8.635090 0.639380
 7.985090 0.489380
 6.251760 0.506050
 6.001760 0.822720
 5.418420 2.172720
 5.935090 2.322720
 6.018420 2.556050
 5.535090 3.156050
 7.718420 1.889380
 6.151760 3.222720
 5.801760 3.822720
 6.235090 3.489380
 8.285090 2.156050
 8.335090 2.422720
 9.851760 2.572720
 10.018420 2.789380
 NA  NA
ID=chilensis


LM=30
 4.032410 6.152760
 3.237970 6.824980
 3.971300 7.374980
 4.093520 7.894420
 4.062970 8.199980
 6.110190 7.680530
 10.265740 8.108310
 11.151860 8.077760
 15.062970 7.436090
 15.918520 6.794420
 14.176860 4.502760
 13.199080 4.105530
 12.343520 3.861090
 11.365740 3.616650
 10.357410 3.463870
 8.401860 3.463870
 8.310190 3.647200
```

```
 7.454630 5.541650
 8.157410 5.694420
 8.218520 5.908310
 7.668520 6.672200
 10.296300 4.930530
 8.524080 6.641650
 8.035190 7.405540
 8.615740 6.977760
 10.968520 5.327760
 11.090740 5.724980
 13.076860 5.786090
 13.229630 5.999980
 NA  NA
ID=chillan


LM=30
 3.510770 4.096710
 3.071120 5.063940
 3.884470 5.305740
 3.994390 5.789360
 3.928440 6.053140
 4.060330 5.393670
 10.149460 6.338920
 12.039950 6.382880
 15.469210 5.745390
 16.348510 5.239790
 14.611900 2.228200
 13.886480 1.788550
 13.007180 1.348910
 11.556340 0.887280
 10.325320 0.799350
 7.995190 0.755380
 7.731400 1.238990
 7.159860 3.261380
 7.775360 3.283360
 7.863290 3.591110
 7.335710 4.448430
 10.061530 2.689830
 8.324920 4.580320
 7.929240 5.415650
 8.456820 4.954020
 11.116690 2.931640
 11.226600 3.547150
 12.919250 3.920850
 13.161060 4.250590
 12.721410 4.558340
ID=chubutensis


LM=30
 3.782420 3.846320
 3.170760 4.605610
 4.119880 5.048530
 4.056610 5.470360
 3.972240 5.828920
 6.060300 5.343810
```

```
 10.826990 5.892190
 11.923750 5.765650
 15.741320 5.238360
 16.774800 4.647790
 15.150750 2.285540
 14.117270 1.737160
 13.273610 1.420790
 12.050300 1.104410
 11.101180 0.872410
 8.907660 0.745860
 8.654560 1.083320
 7.810900 3.002650
 8.422550 3.171390
 8.506920 3.340120
 7.831990 4.204870
 10.805900 2.580820
 8.654560 4.247050
 8.338190 5.132900
 8.844380 4.647790
 11.565190 3.044840
 11.670650 3.403390
 13.779800 3.382300
 13.948540 3.656490
 NA  NA
ID=coquimbo


LM=30
 3.704110 3.427270
 2.808420 4.139740
 3.602320 4.852220
 3.826240 5.259340
 3.744820 5.686830
 5.861890 5.198270
 10.014590 5.707180
 11.174910 5.727540
 15.083340 5.238990
 16.080800 4.669010
 14.228370 2.205880
 13.454830 1.798750
 12.518430 1.411980
 11.297050 1.126990
 10.421720 0.882710
 8.243590 0.638440
 7.958600 0.903070
 7.164700 2.938710
 7.734680 3.101560
 7.816100 3.345840
 7.347900 4.078670
 10.075660 2.450160
 8.101090 4.221170
 7.632890 5.015070
 8.223230 4.526510
 10.828850 2.959070
 10.910280 3.366200
 12.660930 3.752970
 12.823780 3.936180
```

```
 NA  NA
ID=cumelafquen


LM=30
 NA  NA
 2.309990 6.372670
 2.828180 6.804490
 2.943340 7.063590
 2.885760 7.293890
 4.411540 6.919650
 7.319160 6.948430
 8.096450 6.948430
 11.176800 6.286300
 11.954080 5.825690
 10.601030 4.012030
 9.996470 3.781720
 9.161610 3.551410
 8.269180 3.465050
 7.635830 3.436260
 5.994900 3.666560
 5.793380 3.954450
 5.361560 5.336290
 5.822170 5.393860
 5.937320 5.480230
 5.534290 6.056000
 7.520680 4.702940
 6.110050 6.027210
 5.879740 6.574180
 6.282780 6.199940
 8.067660 4.846890
 8.240390 5.105980
 9.622230 5.163560
 9.679800 5.278710
 NA  NA
ID=delpontei_sepiapes


LM=30
 2.702150 2.884090
 2.219700 3.349900
 2.652240 3.765810
 2.768690 4.098530
 2.718780 4.381340
 4.033040 4.031980
 7.293720 4.481160
 7.892620 4.547700
 11.253120 4.131800
 12.084930 3.616080
 10.737400 1.719560
 10.138500 1.336930
 9.323330 1.037480
 8.574700 0.771300
 7.726260 0.638210
 6.212370 0.521760
 6.012740 0.837850
 5.447110 2.251920
```

```
 5.879650 2.385010
 5.946190 2.568000
 5.413840 3.216810
 7.692990 1.969100
 5.996100 3.166900
 5.646740 3.915530
 6.079280 3.516260
 8.158800 2.251920
 8.175440 2.617910
 9.589510 2.834180
 9.689330 2.950640
 9.506330 3.083720
ID=diaguita


LM=30
 2.352470 6.440360
 1.777830 7.151810
 2.434560 7.644360
 2.571370 8.027440
 2.598740 8.328440
 4.185820 7.753810
 8.235620 8.000080
 9.248070 8.000080
 13.434690 7.370720
 14.337690 6.768720
 12.449610 4.497550
 11.847610 4.169190
 10.944610 3.813460
 9.904800 3.594550
 8.864980 3.457740
 6.703270 3.403010
 6.429630 3.813460
 5.800270 5.646820
 6.511720 5.728910
 6.593810 5.865730
 5.937090 6.686630
 8.673440 5.044820
 6.785360 6.796090
 6.539080 7.343360
 6.949540 7.042360
 9.412250 5.400550
 9.494350 5.728910
 10.999340 6.002540
 11.108790 6.166720
 NA  NA
ID=elquiensis


LM=30
 NA  NA
 2.098430 3.776590
 2.762480 4.030490
 2.782010 4.460170
 2.821080 4.772670
 4.383560 4.225800
 7.899160 4.811730
```

```
 9.246800 4.850790
 12.664740 4.499240
 13.504570 4.128140
 11.844430 2.253160
 11.160840 1.862540
 10.360070 1.530510
 9.305390 1.198480
 8.446030 0.944580
 6.590570 0.710210
 6.336670 1.042240
 5.594490 2.409410
 6.297610 2.663310
 6.356200 2.878160
 5.887450 3.444560
 8.348370 2.331290
 6.629640 3.561740
 6.199950 4.108610
 6.707760 3.757050
 8.895240 2.663310
 8.953830 2.975810
 10.477260 3.229720
 10.653040 3.522680
 10.555380 3.620340
ID=erynnis


LM=30
 NA  NA
 2.914490 4.297960
 3.717800 4.807380
 3.913730 5.199240
 3.698210 5.532320
 5.657510 4.983720
 9.850390 5.258020
 11.025970 5.258020
 15.042520 4.572270
 15.982980 3.808140
 14.180430 1.672510
 13.318340 1.300250
 12.181950 0.967170
 10.947600 0.751640
 9.967950 0.634090
 7.871510 0.673270
 7.655990 0.986760
 6.872270 2.887270
 7.538430 2.926460
 7.675580 3.141980
 7.185760 3.906110
 9.654470 2.319080
 8.106620 3.925700
 7.773540 4.591860
 8.204590 4.317560
 10.536150 2.671750
 10.594930 3.063610
 12.593410 3.083200
 12.769740 3.318320
 12.573810 3.455470
```

```
ID=fairchildi


LM=30
 5.457450 6.893610
 5.060880 7.346830
 5.400790 7.686750
 5.655730 8.026660
 5.655730 8.253280
 6.987070 7.941680
 9.876370 8.309930
 10.584530 8.309930
 13.728770 7.885030
 14.408600 7.375160
 13.020610 5.732220
 12.482400 5.448960
 11.830900 5.165690
 10.924450 4.882430
 10.131310 4.740800
 8.573360 4.684140
 8.488380 4.825780
 7.865190 6.270430
 8.346740 6.327080
 8.403400 6.553690
 8.120130 7.091890
 10.046330 6.072140
 8.714990 7.148550
 8.488380 7.715070
 8.771640 7.403480
 10.471230 6.383730
 10.556210 6.553690
 11.887550 6.751970
 11.944200 6.921930
 11.717590 7.120220
ID=fornesi


LM=30
 NA  NA
 2.904720 4.001100
 3.546030 4.605760
 3.527710 5.063840
 3.472740 5.393660
 5.433320 5.045520
 9.207890 5.631860
 10.417220 5.686830
 14.393340 5.283720
 15.419440 4.770670
 13.825330 2.516920
 13.110720 2.040520
 12.212890 1.619090
 11.150140 1.215980
 10.179020 0.977780
 7.851980 0.629640
 7.797010 0.977780
 7.045760 2.938360
 7.577130 2.993330
```

```
 7.650420 3.304820
 7.137370 3.964450
 9.812550 2.590220
 7.778680 4.092720
 7.448870 4.843960
 7.888620 4.477500
 10.527160 3.084940
 10.673740 3.433080
 12.451090 3.616310
 12.634320 3.854510
 NA  NA
ID=frequens


LM=30
 2.672460 2.659780
 2.291100 3.147910
 2.718220 3.559780
 2.702970 3.819100
 2.702970 4.078420
 3.938560 3.758080
 6.775850 4.215710
 7.736870 4.291980
 10.360600 4.124190
 11.077550 3.712320
 10.040260 1.744520
 9.292800 1.363160
 8.667380 1.088590
 7.950430 0.905540
 7.386020 0.753000
 5.906360 0.661470
 5.647040 0.920790
 5.052120 2.293670
 5.540260 2.415710
 5.647040 2.568250
 5.235170 2.995370
 6.989410 2.080120
 5.830090 3.117410
 5.433480 3.605540
 5.845340 3.346220
 7.477550 2.354690
 7.492800 2.614010
 8.636870 2.873340
 8.743650 3.163170
 8.377550 3.239440
ID=hepperi


LM=30
 NA  NA
 2.694000 5.465780
 3.171540 6.034280
 3.330710 6.489070
 3.398930 6.830160
 5.013450 6.193450
 9.652350 6.557290
 11.107690 6.511810
```

```
 15.814810 5.556740
 16.883580 4.692630
 14.746040 1.850170
 13.813720 1.440850
 12.813170 1.122500
 11.607960 0.872360
 10.493720 0.622230
 7.969610 0.599490
 7.719480 1.099760
 6.946330 3.532910
 7.628520 3.646610
 7.742220 3.896740
 6.923590 4.897290
 10.107140 2.759760
 7.764960 4.897290
 7.332900 5.852360
 7.924130 5.329340
 10.880290 3.260030
 10.903030 3.805780
 13.154260 3.692090
 13.267960 3.919480
 NA  NA
ID=hirsuta


LM=30
 3.374590 3.053320
 2.750210 3.753380
 3.279990 4.302080
 3.525950 4.775090
 3.488110 5.077820
 5.474770 4.756170
 9.788660 5.380550
 10.715770 5.437310
 14.670170 5.039980
 15.672960 4.453440
 14.140400 2.201890
 13.326810 1.672120
 12.475390 1.274780
 11.302310 0.858530
 10.280600 0.555800
 8.237180 0.423360
 7.953370 0.688250
 7.045180 2.599220
 7.877690 2.693830
 7.934450 2.901950
 7.139780 3.734460
 10.053550 2.277570
 8.142570 3.791220
 7.574960 4.585880
 8.123650 4.131790
 10.791460 2.674910
 10.867140 2.996550
 12.910560 3.337130
 13.024080 3.488490
 NA  NA
ID=inata
```

```
LM=30
 2.875220 2.932330
 2.378700 3.677110
 3.105740 3.996300
 3.105740 4.386420
 3.034810 4.670140
 4.825820 4.102690
 8.638350 4.616940
 10.234290 4.634670
 13.124730 4.084960
 14.029100 3.482050
 12.379950 1.655580
 11.723840 1.300920
 10.837210 0.928540
 9.737780 0.680280
 8.868880 0.502950
 6.847350 0.449750
 6.599090 0.804410
 6.049380 2.293960
 6.758680 2.364890
 6.847350 2.524480
 6.368560 3.304720
 8.620620 1.974770
 7.219730 3.340190
 6.811880 4.014030
 7.255200 3.588440
 9.365390 2.293960
 9.489520 2.559950
 10.872670 2.896870
 11.032270 3.109660
 10.890410 3.180590
ID=kroeberi


LM=30
 2.639420 6.308060
 2.191260 6.781110
 2.539830 7.129670
 2.763900 7.453340
 2.739000 7.702310
 3.784690 7.353750
 6.971550 7.926390
 7.842960 7.901490
 10.755950 7.453340
 11.428170 7.179470
 10.432280 5.411760
 9.859640 5.063200
 9.087820 4.714630
 8.340900 4.490560
 7.818060 4.341170
 6.249530 4.191790
 6.050350 4.390970
 5.178940 5.760320
 5.726680 5.884810
 5.776480 6.059090
```

```
 5.228740 6.581930
 7.369910 5.561140
 5.751580 6.681520
 5.303430 7.229260
 5.776480 6.955390
 7.842960 5.785220
 7.892750 6.059090
 9.112720 6.382750
 9.287000 6.581930
 9.137620 6.631730
ID=mendozana


LM=30
 2.182480 5.878690
 1.691060 6.717000
 2.182480 7.063890
 2.413740 7.468590
 2.355930 7.757660
 3.627850 7.208420
 7.183460 7.642030
 8.050680 7.584220
 11.461750 7.034980
 12.242250 6.485740
 11.259390 4.722390
 10.710160 4.433320
 9.929660 4.144240
 8.917900 3.826260
 8.050680 3.652820
 6.345140 3.566100
 5.969350 3.797360
 5.102130 5.358350
 5.738090 5.387260
 5.853720 5.647430
 5.275570 6.341200
 7.645980 5.011470
 5.882630 6.341200
 5.477920 6.977170
 5.911530 6.601370
 8.426470 5.387260
 8.455380 5.531800
 9.785120 5.791960
 9.929660 6.109940
 9.785120 6.254480
ID=minor


LM=30
 2.226440 4.908000
 1.733900 5.427910
 2.363260 5.920450
 2.664260 6.303540
 2.554800 6.604540
 4.087160 6.112000
 7.972780 6.495090
 8.437960 6.440360
 12.952940 6.084630
```

```
 13.746490 5.564730
 12.132040 3.485100
 11.420590 3.211460
 10.544950 2.910460
 9.395690 2.691560
 8.520050 2.554740
 6.659330 2.554740
 6.385700 2.910460
 5.427970 4.388100
 6.248880 4.552280
 6.330970 4.607000
 5.893150 5.209000
 8.109600 3.922920
 6.686700 5.263730
 6.248880 5.893090
 6.714060 5.510000
 8.903140 4.196550
 8.957870 4.524910
 10.544950 4.853280
 10.763860 5.017460
 10.544950 5.126910
ID=missionum


LM=30
 NA  NA
 1.771260 6.074810
 2.217270 6.280660
 2.285880 6.520820
 2.320190 6.726670
 3.555280 6.349280
 6.505770 6.520820
 7.260550 6.589430
 10.965820 6.109120
 11.789210 5.628810
 10.279660 3.810480
 9.593500 3.536020
 8.701490 3.364480
 7.843790 3.261550
 7.054700 3.192940
 5.510840 3.330170
 5.304990 3.673250
 4.824680 4.942650
 5.373610 4.942650
 5.407910 5.011260
 4.961910 5.628810
 7.020390 4.290790
 5.853920 5.560190
 5.476530 6.109120
 5.922540 5.800350
 7.672250 4.565260
 7.775170 4.805410
 9.147490 4.976960
 9.319030 5.182800
 9.078880 5.388650
ID=nigra
```

```
LM=30
 4.915570 5.011470
 4.597580 5.445080
 5.031190 5.734150
 5.060100 5.936500
 5.002290 6.138850
 6.389840 5.878690
 8.760240 6.081040
 9.280580 6.109940
 12.142410 5.878690
 12.778370 5.473980
 11.766610 4.086430
 11.275180 3.941890
 10.639220 3.739540
 9.916540 3.566100
 9.280580 3.508280
 7.864120 3.508280
 7.575040 3.768450
 7.228150 4.693480
 7.632860 4.838020
 7.661760 4.924740
 7.343780 5.271630
 9.222760 4.404410
 7.921930 5.329450
 7.632860 5.705240
 7.950840 5.502890
 9.627460 4.635670
 9.685280 4.780210
 10.668130 5.011470
 10.697040 5.098190
 NA  NA
ID=nigrifrons


LM=30
 1.603120 6.184990
 1.198630 7.101840
 1.764920 7.533290
 2.034580 7.937780
 2.034580 8.288340
 3.787360 7.560260
 7.994060 7.722060
 9.261460 7.641160
 13.333320 6.508590
 14.142300 5.726570
 12.254680 3.434470
 11.310870 3.191770
 10.259200 2.868180
 8.937860 2.679420
 8.128880 2.571560
 5.755880 2.760320
 5.540150 3.245700
 5.189590 5.106360
 5.863740 5.106360
 5.998570 5.268150
 5.270490 6.373760
```

```
 7.913160 4.351310
 6.214300 6.319820
 5.836780 7.128800
 6.349130 6.643420
 8.695170 4.513100
 8.856970 4.971530
 10.960310 4.971530
 11.122110 5.214220
 NA  NA
ID=antillanca


LM=30
 3.512210 3.175980
 2.918210 3.813990
 3.600210 4.363980
 3.886210 4.869980
 3.798210 5.265980
 5.712210 4.649990
 9.562210 5.463990
 10.596210 5.419980
 14.446210 4.935980
 15.414210 4.363980
 13.720210 2.251980
 12.972210 1.855980
 12.048210 1.503980
 10.904210 1.151990
 10.178210 0.953990
 8.132210 0.689990
 7.846210 1.041980
 6.944210 2.581980
 7.626210 2.779980
 7.648210 3.087990
 7.186210 3.725990
 10.090210 2.471990
 8.110210 3.923990
 7.516210 4.627980
 8.132210 4.165990
 10.596210 2.801990
 10.640210 3.241990
 12.400210 3.417980
 12.576210 3.659980
 NA  NA
ID=opaca


LM=30
 3.214480 3.341920
 2.717700 3.785470
 3.249960 4.246760
 3.249960 4.672570
 3.498350 4.938700
 5.183830 4.601600
 9.158030 5.133860
 9.725770 5.169340
 13.238670 4.725790
 14.108030 4.211280
```

```
 13.203190 1.958050
 12.387060 1.496760
 11.322540 1.141920
 10.364480 0.858050
 9.406410 0.662890
 7.508030 0.733860
 7.259640 0.999990
 6.603190 2.667730
 7.419320 2.720960
 7.490290 2.898380
 6.833830 3.643540
 9.441900 2.046760
 7.756410 3.714500
 7.153190 4.441920
 7.845120 4.051600
 10.133830 2.454830
 10.169320 2.862890
 11.641900 3.253210
 11.819320 3.537080
 11.588670 3.625800
ID=ornatissima


LM=30
 4.218510 2.955990
 3.558510 3.681990
 4.174510 4.099990
 4.306510 4.627980
 4.174510 4.935980
 6.110510 4.561980
 9.960510 5.617990
 10.884510 5.749980
 15.108510 5.683980
 16.362510 5.221990
 14.954510 2.779980
 14.030510 2.163980
 13.062510 1.745980
 11.874510 1.283980
 11.038510 1.041980
 8.992510 0.755990
 8.574510 1.129990
 7.826510 2.493980
 8.530510 2.779980
 8.574510 3.087990
 7.936510 3.725990
 10.642510 2.625990
 8.552510 3.945980
 8.244510 4.627980
 8.728510 4.363980
 11.346510 3.065980
 11.390510 3.417980
 13.106510 3.813990
 13.260510 4.121980
 NA  NA
ID=pallipes
```

```
LM=30
 3.447270 3.637990
 2.765270 4.451980
 3.601270 4.979990
 3.843270 5.375990
 3.777270 5.639990
 5.625270 5.243990
 9.783270 5.485990
 10.487270 5.485990
 14.931270 5.045980
 15.965270 4.495990
 14.557270 2.163980
 13.655270 1.635990
 12.423270 1.151990
 11.213270 0.909990
 10.025270 0.667990
 8.089270 0.711990
 7.847270 1.085980
 6.945270 3.087990
 7.803270 3.087990
 7.979270 3.263980
 7.341270 4.099990
 10.069270 2.515990
 8.309270 4.143980
 8.133270 4.847990
 8.441270 4.429990
 10.927270 2.933980
 11.015270 3.285990
 12.797270 3.483990
 12.973270 3.659980
 NA  NA
ID=penai


LM=30
 2.723170 3.526260
 2.378810 4.337960
 2.969130 4.731500
 3.116710 5.248030
 3.141310 5.567790
 4.838490 4.879080
 8.749370 5.198840
 9.388880 5.198840
 13.693310 4.583920
 14.677180 3.919810
 12.660250 1.607720
 11.971540 1.238760
 10.938480 0.845220
 9.880820 0.623850
 8.847760 0.451670
 6.732430 0.451670
 6.634050 0.722230
 6.043730 2.689970
 6.781630 2.763760
 6.880020 3.034330
 6.338890 3.846020
 8.847760 2.075050
```

```
 7.125980 3.870620
 6.806230 4.608520
 7.248970 4.141180
 9.536460 2.296430
 9.831630 2.714570
 11.405820 3.009730
 11.602590 3.255700
 NA  NA
ID=pereirai


LM=30
 3.479890 3.944830
 2.958230 4.798470
 3.622170 5.249000
 3.977850 5.723240
 3.977850 6.055210
 5.613990 5.391270
 10.048170 5.770670
 10.901810 5.628390
 15.762810 4.656200
 16.829850 3.849980
 14.577200 1.597320
 13.557570 1.289060
 12.585370 0.980810
 11.257490 0.719970
 10.095590 0.553990
 7.724380 0.506560
 7.510970 0.957090
 7.013010 2.996340
 7.724380 3.020050
 7.842940 3.257180
 7.392410 4.229380
 10.048170 2.284980
 8.293470 4.229380
 7.795510 5.083010
 8.317180 4.585060
 10.949230 2.522100
 11.138930 2.925210
 13.012190 3.091190
 13.201890 3.304600
 13.012190 3.423160
ID=pereirai_dureti


LM=30
 3.149630 3.323140
 2.691550 3.872840
 3.222920 4.275950
 3.424480 4.642410
 3.369510 4.917260
 5.494990 4.422530
 8.591610 5.008870
 9.782610 5.027200
 13.099100 4.917260
 13.941970 4.459180
 12.421150 2.333690
```

```
 11.761510 1.893940
 10.973620 1.564120
 10.020810 1.197660
 9.177950 0.941130
 7.400600 0.702930
 7.089110 1.069390
 6.301210 2.498600
 6.924200 2.681830
 6.979170 2.938360
 6.557740 3.616310
 8.958070 2.352020
 7.308990 3.652960
 6.905880 4.275950
 7.418920 3.909480
 9.654350 2.553570
 9.819260 2.956680
 11.285110 3.304820
 11.504990 3.634640
 NA  NA
ID=persignata


LM=30
 3.515930 3.308490
 2.859210 4.038180
 3.461210 4.512480
 3.716600 4.986780
 3.607140 5.242170
 5.431380 4.840840
 9.426450 5.424600
 9.900750 5.406360
 13.822860 4.913810
 14.589040 4.348300
 13.366800 2.068010
 12.618860 1.630190
 11.688500 1.228860
 10.593960 1.028190
 9.663600 0.791040
 7.711670 0.663350
 7.419790 1.101160
 6.744830 2.834190
 7.365070 2.925400
 7.456280 3.162550
 7.000220 3.801030
 9.371730 2.451100
 7.675190 3.928730
 7.328580 4.694910
 7.748160 4.384780
 10.192630 2.742970
 10.210870 3.144310
 11.889170 3.417940
 12.089840 3.636850
 NA  NA
ID=pruinivitta


LM=30
```

```
 2.012700 5.849780
 1.376740 6.572460
 2.099420 7.179520
 2.359590 7.670940
 2.330680 7.960020
 4.672180 7.295150
 9.066100 7.613130
 10.569280 7.526400
 14.905380 6.717000
 15.743700 6.138850
 13.633460 3.623910
 12.679520 3.305930
 11.523220 3.045760
 10.309110 2.872320
 9.326260 2.727780
 6.869140 2.785600
 6.695690 3.045760
 5.799560 5.098190
 6.637880 5.156000
 6.695690 5.300540
 6.088640 6.167760
 9.846590 4.664580
 7.273840 6.052130
 6.695690 6.919350
 7.331650 6.427930
 10.164580 4.693480
 10.193480 5.040370
 12.332630 5.098190
 12.477160 5.300540
 NA  NA
ID=senilis


LM=30
 2.663650 7.785400
 2.056270 8.483880
 2.694010 8.969780
 2.906590 9.364570
 2.845860 9.637890
 4.728720 9.151990
 8.798120 9.425310
 9.648440 9.455680
 14.112630 8.726830
 15.023690 8.149830
 13.171210 5.811440
 12.411990 5.447020
 11.470560 5.112960
 10.073600 4.839640
 9.132170 4.718170
 7.097470 4.718170
 6.915260 5.052220
 6.186410 6.965450
 6.854520 6.995820
 6.976000 7.238770
 6.398990 7.997980
 9.010700 6.358080
 7.249310 7.997980
```

```
 6.763420 8.817940
 7.340420 8.332040
 9.678810 6.661760
 9.891390 7.026180
 11.804620 7.299500
 11.956460 7.542450
 11.531300 7.785400
ID=shannoni


LM=30
 3.333830 3.455470
 2.824420 3.984480
 3.353430 4.533080
 3.431800 4.924940
 3.412200 5.218830
 5.018830 4.807380
 8.761080 5.414760
 9.701540 5.395170
 13.287050 4.650640
 13.992390 4.141220
 12.522920 1.829260
 11.915540 1.476580
 11.151420 1.182690
 10.250140 0.849610
 9.348870 0.692860
 7.389570 0.673270
 7.115270 1.163090
 6.429520 2.769720
 7.115270 2.828500
 7.154460 3.004830
 6.468700 3.808140
 9.054970 2.299480
 7.115270 3.925700
 6.801780 4.650640
 7.213230 4.239190
 9.721130 2.691340
 9.819100 3.024420
 11.229790 3.357500
 11.347350 3.592620
 11.053450 3.749360
ID=subtrita


LM=30
 4.266610 8.505660
 3.550310 9.290180
 4.198390 9.767710
 4.437160 10.245250
 4.437160 10.756890
 6.620170 9.938260
 11.600160 10.620450
 13.066880 10.552230
 17.773990 9.733600
 18.763170 8.914970
 16.648380 6.186210
 15.659200 5.776890
```

```
 14.397150 5.333470
 12.998660 5.026490
 11.838930 4.855940
 9.417150 4.821830
 9.212500 5.094700
 8.257430 7.516480
 9.076060 7.584700
 9.178390 7.857580
 8.462090 8.812640
 11.566050 6.868400
 9.451260 8.914970
 9.212500 9.835930
 9.724140 9.221960
 12.555230 7.209500
 12.725780 7.721140
 14.772350 7.994020
 15.079340 8.266890
 NA  NA
ID=testaceomaculata


LM=30
 3.088160 3.265760
 2.617030 3.835040
 3.127420 4.247280
 3.245200 4.639890
 3.264830 4.914710
 5.011930 4.482840
 8.643550 5.111010
 9.526910 5.150270
 13.276310 4.698780
 14.061520 4.070610
 12.589250 1.989790
 11.882550 1.577550
 11.038450 1.322360
 10.115820 1.047530
 9.232460 0.870860
 7.249790 0.733450
 7.053490 1.027900
 6.052340 2.735740
 6.680510 2.892780
 6.798300 3.049830
 6.366430 3.638740
 8.780960 2.421660
 7.151640 3.736890
 6.759030 4.384690
 7.210530 4.031340
 9.526910 2.696480
 9.664330 2.971310
 11.018820 3.363910
 11.136600 3.619110
 10.901040 3.658370
ID=trigonophora


LM=30
 4.388470 6.326340
```

```
 3.869830 6.875490
 4.388470 7.302610
 4.693560 7.729730
 4.571520 8.004300
 5.974920 7.516170
 9.452890 7.912780
 10.886790 7.882270
 13.693570 7.272100
 14.425770 6.783960
 12.961370 4.648370
 12.259670 4.312770
 11.527470 4.068700
 10.612210 3.916160
 9.818990 3.794130
 7.988480 3.885650
 7.652880 4.312770
 7.195260 5.563620
 7.713900 5.655150
 7.805430 5.868710
 7.408820 6.509390
 9.574920 5.167010
 7.988480 6.600910
 7.774920 7.272100
 8.110510 6.875490
 10.154580 5.441590
 10.276620 5.746670
 11.771530 5.990740
 11.954580 6.204300
 11.680010 6.356840
ID=tritus


LM=30
 3.595380 3.756860
 3.040050 4.589860
 3.687930 5.191460
 3.896180 5.654230
 3.942460 5.955040
 6.024940 5.492260
 10.074210 6.232700
 10.837780 6.209560
 15.442380 5.677370
 16.414200 5.145180
 14.887050 2.252850
 14.100340 1.836350
 13.174790 1.443000
 11.902160 1.026500
 10.768370 0.795110
 8.570190 0.656280
 8.269390 1.049640
 7.297570 3.039570
 8.107420 3.155260
 8.269390 3.409780
 7.737200 4.358470
 10.490700 2.692490
 8.570190 4.497300
 7.991730 5.353430
```

```
 8.616470 4.798100
 11.277420 3.108980
 11.369970 3.525480
 13.244200 3.872560
 13.383040 4.150220
 12.758290 4.497300
ID=vasta


LM=30
 5.125430 5.406470
 4.760410 5.849700
 5.203650 6.162570
 5.203650 6.423300
 5.177570 6.657950
 6.637630 6.240790
 9.088450 6.710100
 10.209570 6.736170
 12.842890 6.319010
 13.442560 5.953990
 11.826060 4.624300
 11.408900 4.181060
 10.809230 3.894270
 10.131350 3.633540
 9.505610 3.451030
 7.758750 3.503180
 7.654460 3.685690
 7.419810 4.728590
 7.836970 4.885020
 7.889110 5.067530
 7.498030 5.615050
 9.349170 4.520010
 8.045550 5.615050
 7.810900 6.188650
 8.097690 5.849700
 9.974910 4.832880
 10.000990 5.093600
 11.174250 5.223960
 11.382830 5.406470
 NA  NA
ID=collagua


LM=30
 3.531200 6.327710
 2.741610 7.117290
 3.318620 7.694300
 3.713410 8.210560
 3.865250 8.726830
 5.444420 7.967610
 10.333780 8.635730
 11.912950 8.635730
 15.648300 8.180190
 16.620090 7.572820
 14.889080 4.809270
 14.129860 4.323370
 12.975850 3.837470
```

```
 11.670000 3.503420
 10.698200 3.230100
 8.511660 2.926410
 8.299080 3.321210
 7.418390 5.416650
 8.147240 5.568490
 8.268710 5.811440
 7.448760 6.752870
 10.607100 5.021850
 8.420550 6.783240
 7.843550 7.694300
 8.481290 7.208400
 11.366310 5.416650
 11.518160 5.872180
 13.309910 6.266970
 13.461750 6.509920
 13.066960 6.692130
ID=Agelanius


LM=30
 3.716930 5.986690
 3.006390 6.923310
 3.684630 7.472360
 3.910710 8.021410
 3.910710 8.505860
 6.106920 7.698430
 10.886890 8.441270
 11.662020 8.441270
 16.506590 7.633840
 17.636980 6.891010
 15.925240 4.307240
 14.924030 3.725890
 13.664440 3.273730
 12.307960 2.886170
 10.951480 2.692380
 8.755280 2.434010
 8.626090 2.660090
 7.721770 5.017780
 8.496900 5.211560
 8.626090 5.566830
 7.786370 6.568040
 11.112970 4.565620
 8.884470 6.632630
 8.432310 7.504650
 9.045950 6.955600
 12.081880 5.017780
 12.211070 5.469940
 14.181190 5.857500
 14.374980 6.212770
 NA  NA
ID=Acellomyia


LM=30
 5.349720 7.491610
 4.938020 8.006230
```

```
 5.349720 8.383620
 5.521260 8.692390
 5.452650 9.069780
 7.065120 8.623780
 10.221470 9.035470
 10.941930 9.069780
 14.509970 8.589470
 15.401980 8.040540
 14.063970 6.153600
 13.069030 5.638970
 12.348560 5.501740
 11.456560 5.227280
 10.598850 5.124350
 8.780530 5.124350
 8.677600 5.433130
 8.094370 6.736830
 8.780530 6.839760
 8.849140 7.079910
 8.368830 7.697460
 10.358700 6.462370
 8.952070 7.766070
 8.814840 8.383620
 9.123610 8.040540
 11.113470 6.736830
 11.147780 7.114220
 12.314260 7.388680
 12.554410 7.628840
 NA  NA
ID=Haematopotina


LM=30
 6.750050 9.620210
 6.420250 10.321020
 6.873720 10.733270
 6.997390 11.104280
 6.956170 11.516530
 8.481470 10.939390
 12.438990 11.598980
 13.345930 11.598980
 17.550800 10.774490
 18.540180 9.991230
 17.056110 7.723900
 16.190400 7.270430
 15.407140 7.064310
 14.458980 6.816960
 13.510820 6.610840
 11.325940 6.734510
 11.078590 7.064310
 10.130440 8.836950
 10.790020 9.043070
 10.872470 9.331640
 10.212880 9.950010
 12.851240 8.713280
 10.872470 10.073680
 10.336560 10.774490
 10.913700 10.321020
```

```
 13.469600 8.836950
 13.510820 9.084300
 14.871220 9.455310
 15.077340 9.785110
 NA  NA
ID=Stenotabanus_sandyi


LM=30
 2.852150 5.420710
 2.412700 6.134820
 2.879620 6.491870
 3.236670 7.041180
 3.209210 7.315840
 4.719820 6.656670
 8.592480 7.233440
 9.608710 7.178510
 12.932060 6.601730
 13.701100 6.024960
 12.492610 4.157290
 11.778500 3.772770
 10.927070 3.305850
 9.800970 2.948800
 9.031930 2.729070
 6.917080 2.481880
 6.669880 2.729070
 5.900850 4.596740
 6.560020 4.761530
 6.697350 5.036190
 6.202970 5.722830
 8.647410 4.267150
 6.999470 5.832700
 6.560020 6.491870
 7.026940 6.024960
 9.388990 4.569270
 9.443920 4.926330
 10.872130 5.255920
 11.064390 5.612970
 10.707340 5.832700
ID=dubiosa


LM=30
 1.826570 3.205540
 1.500650 3.755540
 1.948790 4.061100
 2.132130 4.407390
 2.071020 4.631470
 3.272870 4.183320
 6.206200 4.427760
 6.817310 4.427760
 9.608050 4.101840
 10.300650 3.694430
 9.343240 2.044430
 8.630280 1.637020
 7.835830 1.351840
 7.041390 1.168500
```

```
 6.491390 1.066650
 5.024720 1.005540
 4.861760 1.209250
 4.250650 2.635170
 4.800650 2.757390
 4.841390 2.940730
 4.474720 3.409250
 6.389540 2.248130
 5.146940 3.449990
 4.882130 3.979620
 5.269170 3.592580
 6.776570 2.512950
 6.817310 2.798130
 7.998790 2.981470
 8.182130 3.225910
 8.059910 3.307390
ID=dixoni


LM=30
 3.026340 6.471780
 2.752710 7.046410
 2.971610 7.347410
 3.081070 7.675770
 3.108430 7.922040
 4.476610 7.621050
 7.869680 8.359860
 8.909500 8.469310
 12.439390 8.332500
 13.506570 7.757860
 12.494120 5.842420
 11.563760 5.322510
 10.688130 4.994150
 9.839860 4.802600
 9.018950 4.611060
 7.158230 4.474240
 6.884600 4.720510
 5.981600 6.061320
 6.528870 6.307600
 6.583600 6.444410
 6.118420 6.936960
 8.416950 6.006600
 6.665690 7.073770
 6.227870 7.566320
 6.720420 7.292680
 8.936860 6.280230
 8.964220 6.471780
 10.441860 6.882230
 10.523950 7.183230
 10.332400 7.292680
ID=Stenotabanus_taeniotes
```

Supplementary table V

K-value	P-cut	%_supported	%_compatible	%_supportedScaled	%_NcompatibleScaled
0	33	0,237	0,305	0,13	0,173
1	33	0,159	0,16	0,128	0,129
2	33	0,192	0,192	0,155	0,155
3	33	0,163	0,163	0,133	0,133
4	33	0,171	0,173	0,141	0,142
5	33	0,183	0,196	0,151	0,162
6	33	0,196	0,205	0,162	0,17
7	33	0,191	0,204	0,159	0,17
8	33	0,195	0,213	0,162	0,177
9	33	0,181	0,191	0,151	0,159
10	33	0,185	0,19	0,154	0,159
11	33	0,196	0,196	0,164	0,164
12	33	0,199	0,199	0,167	0,167
13	33	0,194	0,194	0,163	0,163
14	33	0,195	0,195	0,165	0,165
15	33	0,197	0,197	0,166	0,166
16	33	0,197	0,197	0,167	0,167
17	33	0,193	0,193	0,164	0,164
18	33	0,196	0,196	0,167	0,167
19	33	0,195	0,195	0,165	0,165
20	33	0,199	0,203	0,169	0,173
21	33	0,2	0,203	0,171	0,173
22	33	0,199	0,205	0,169	0,175
23	33	0,202	0,202	0,172	0,172
24	33	0,208	0,208	0,177	0,177
25	33	0,21	0,21	0,178	0,178
26	33	0,213	0,213	0,182	0,182
27	33	0,212	0,212	0,181	0,181
28	33	0,2	0,2	0,17	0,17
29	33	0,213	0,213	0,182	0,182
30	33	0,209	0,209	0,179	0,179
31	33	0,209	0,209	0,178	0,178
32	33	0,194	0,194	0,165	0,165
33	33	0,191	0,194	0,163	0,165
34	33	0,19	0,194	0,162	0,165
35	33	0,192	0,193	0,164	0,164
36	33	0,193	0,194	0,164	0,166
37	33	0,194	0,195	0,166	0,166
38	33	0,193	0,196	0,165	0,167
39	33	0,19	0,193	0,162	0,165
40	33	0,196	0,196	0,167	0,167
41	33	0,192	0,194	0,163	0,166
42	33	0,192	0,195	0,163	0,166
43	33	0,192	0,193	0,164	0,165
44	33	0,191	0,192	0,163	0,164
45	33	0,189	0,191	0,162	0,163
46	33	0,191	0,196	0,163	0,167
47	33	0,19	0,194	0,162	0,165
48	33	0,192	0,194	0,164	0,165
49	33	0,192	0,194	0,163	0,165
50	33	0,191	0,192	0,163	0,164

51	33	0,19	0,195	0,163	0,167
52	33	0,191	0,194	0,163	0,166
53	33	0,189	0,196	0,162	0,168
54	33	0,192	0,195	0,164	0,167
55	33	0,192	0,195	0,164	0,166
56	33	0,191	0,193	0,163	0,165
57	33	0,208	0,208	0,178	0,178
58	33	0,19	0,195	0,162	0,166
59	33	0,206	0,206	0,176	0,176
60	33	0,209	0,209	0,178	0,178
61	33	0,191	0,191	0,163	0,163
62	33	0,192	0,193	0,164	0,165
63	33	0,188	0,193	0,161	0,165
64	33	0,189	0,194	0,161	0,165
65	33	0,188	0,19	0,16	0,163
66	33	0,189	0,193	0,162	0,165
67	33	0,204	0,204	0,175	0,175
68	33	0,189	0,193	0,161	0,165
69	33	0,19	0,192	0,163	0,164
70	33	0,178	0,179	0,153	0,153

MaxAveagerDist	MiinAveagerDist
0,666	0,559
0,561	0,502
0,599	0,563
0,575	0,52
0,592	0,522
0,62	0,554
0,639	0,576
0,641	0,58
0,654	0,589
0,666	0,624
0,672	0,635
0,673	0,61
0,684	0,616
0,686	0,612
0,687	0,615
0,693	0,619
0,696	0,619
0,693	0,621
0,694	0,624
0,696	0,624
0,695	0,635
0,695	0,629
0,698	0,637
0,698	0,61
0,69	0,677
0,689	0,679
0,689	0,679
0,69	0,681
0,696	0,655
0,688	0,68
0,687	0,679
0,689	0,678
0,685	0,662
0,681	0,66
0,685	0,66
0,682	0,659
0,683	0,66
0,682	0,651
0,684	0,661
0,683	0,658
0,683	0,644
0,682	0,658
0,683	0,658
0,68	0,657
0,681	0,656
0,684	0,657
0,681	0,659
0,683	0,658
0,679	0,656
0,68	0,659
0,683	0,658
0,681	0,659
0,687	0,661
0,685	0,658

0,681	0,658
0,682	0,657
0,68	0,657
0,681	0,653
0,684	0,658
0,682	0,651
0,683	0,652
0,677	0,657
0,684	0,649
0,685	0,657
0,688	0,657
0,681	0,657
0,683	0,659
0,68	0,651
0,682	0,658
0,682	0,654
0,672	0,633
